# Integrated trajectories of systolic and diastolic function differentially associate with risk for heart failure with preserved and reduced ejection fraction and proteomic profiles

**DOI:** 10.1002/ejhf.70015

**Published:** 2025-09-02

**Authors:** Anne Marie Reimer Jensen, James C. Ross, Victoria Arthur, Michael E. Hall, Kunihiro Matsushita, Brandon Lennep, Pamela L. Lutsey, Tor Biering‐Sørensen, Amil M. Shah

**Affiliations:** ^1^ Department of Cardiology Copenhagen University Hospital ‐ Herlev and Gentofte Copenhagen Denmark; ^2^ Division of Cardiovascular Medicine Brigham and Women's Hospital Boston MA USA; ^3^ Center for Translational Cardiology and Pragmatic Randomized Trials, Department of Biomedical Sciences, Faculty of Health and Medical Sciences University of Copenhagen Copenhagen Denmark; ^4^ Department of Radiology Brigham and Women's Hospital Boston MA USA; ^5^ Division of Cardiology University of Texas Southwestern Medical Center Dallas TX USA; ^6^ Division of Cardiovascular Diseases University of Mississippi Medical Center Jackson MS USA; ^7^ Department of Epidemiology Johns Hopkins Bloomberg School of Public Health Baltimore MD USA; ^8^ Division of Epidemiology and Community Health University of Minnesota Minneapolis MN USA

**Keywords:** Heart failure, Echocardiography, Trajectory, Ageing, Epidemiology

## Abstract

**Aims:**

To jointly model longitudinal measures of left ventricular ejection fraction (LVEF) and E/A ratio in late‐life, and to assess whether predicted trajectory membership is associated with heart failure risk.

**Methods and results:**

Using a Bayesian non‐parametric trajectory approach, trajectories were modelled among 747 Jackson Heart Study participants who underwent ≥2 echocardiograms in 2000–2004 (age 65 ± 5 years), 2011–2013 (75 ± 5), and 2018–2019 (81 ± 5). Using the resulting model, we predicted trajectory membership for 4419 distinct Atherosclerosis Risk in Communities (ARIC) study participants based on single time‐point measures of LVEF and E/A ratio (age 75 ± 5 years; ‘testing cohort’). Multivariable Cox models assessed the relationship between predicted trajectory and incident heart failure with preserved (HFpEF) and reduced ejection fraction (HFrEF). We evaluated associations of 4877 plasma proteins (SOMAscan) with predicted trajectory and performed Mendelian randomization to assess causal effects on LVEF and volume. Six trajectories were identified: pink (prevalence 50%) and light green (17%) – increasing LVEF, decreasing E/A ratio with age; red (22%) – no increase in LVEF; dark green (4%) – declining LVEF; orange (2%) – steeply declining LVEF, rising E/A ratio; and blue (4%) – rising E/A ratio despite increasing LVEF. In the testing cohort, red and dark green associated with HFrEF alone, blue with HFpEF alone, and orange with both compared to pink. Trajectory membership provided incremental value in predicting heart failure and HFpEF. Mendelian randomization identified potential causal effects of 13 trajectory‐associated proteins on LVEF and volume.

**Conclusions:**

Bayesian non‐parametric modelling identifies cardiac function trajectories differentially associated with HFpEF and HFrEF and holds promise to improve risk prediction and enable therapeutic target discovery.

## Introduction

Heart failure (HF) incidence and prevalence are rising in concert with the ageing of the population.^1^ Impairments in systolic and diastolic function, and associated alterations in cardiac structure, precede and underlie HF. Age‐related changes in cardiac structure and function are characterized by increasing left ventricular (LV) wall thickness, decreasing LV chamber dimension, and increasing fractional shortening^2^, ^3^ and these changes are modified by known cardiovascular risk factors such that changes in chamber dimension and fractional shortening are abrogated and LV mass increases.^3^ However, large inter‐individual variability exists in the extent and character of cardiac changes through the course of life even after accounting for demographic and cardiovascular risk factors, and likely underlies individual variability in risk of developing HF. Identifying trajectories of cardiac function through mid‐ to late‐life offers promise to identify subpopulations of individuals with distinct progression characteristics and latent risk for HF with preserved and reduced LV ejection fraction (LVEF). Furthermore, the ability to predict an individual's trajectory based on multiple parameters at a single timepoint may improve risk prediction and help identify individuals for targeted prevention efforts.

The goal of trajectory modelling is to identify the number of subgroups, the progression characteristics of each subgroup, and which subgroup each individual in a data sample is most likely to be a member of. Bayesian non‐parametric mixture modelling is a data‐driven approach to trajectory modelling that has previously been used to characterize prototypical lung function trajectories.^4^, ^5^ The advantages of this approach include the possibility of modelling several measures simultaneously to derive multiparametric trajectories, and the absence of an assumption of balanced or time‐structured data such that individuals can differ with respect to the number of longitudinal data points collected. We utilize this novel modelling approach to identify trajectories of cardiac function in mid‐ to late‐life among 747 adults who underwent two or more echocardiograms over an 18‐year period through their participation in both the Jackson Heart Study (JHS) and the Atherosclerosis Risk in Communities (ARIC) study. Then, among a separate set of 4419 participants who underwent protocol echocardiography only in the ARIC study, we (i) predicted trajectory membership, (ii) determined the association of trajectory membership with risk of incident HF with preserved (HFpEF) or reduced (HFrEF) ejection fraction, (iii) identified proteomic measures uniquely associated with each trajectory, and (iv) evaluated potential causality of proteins on LV volume and LVEF through two‐sample Mendelian randomization (MR) analysis.

## Methods

### Study populations

**Figure 1:**
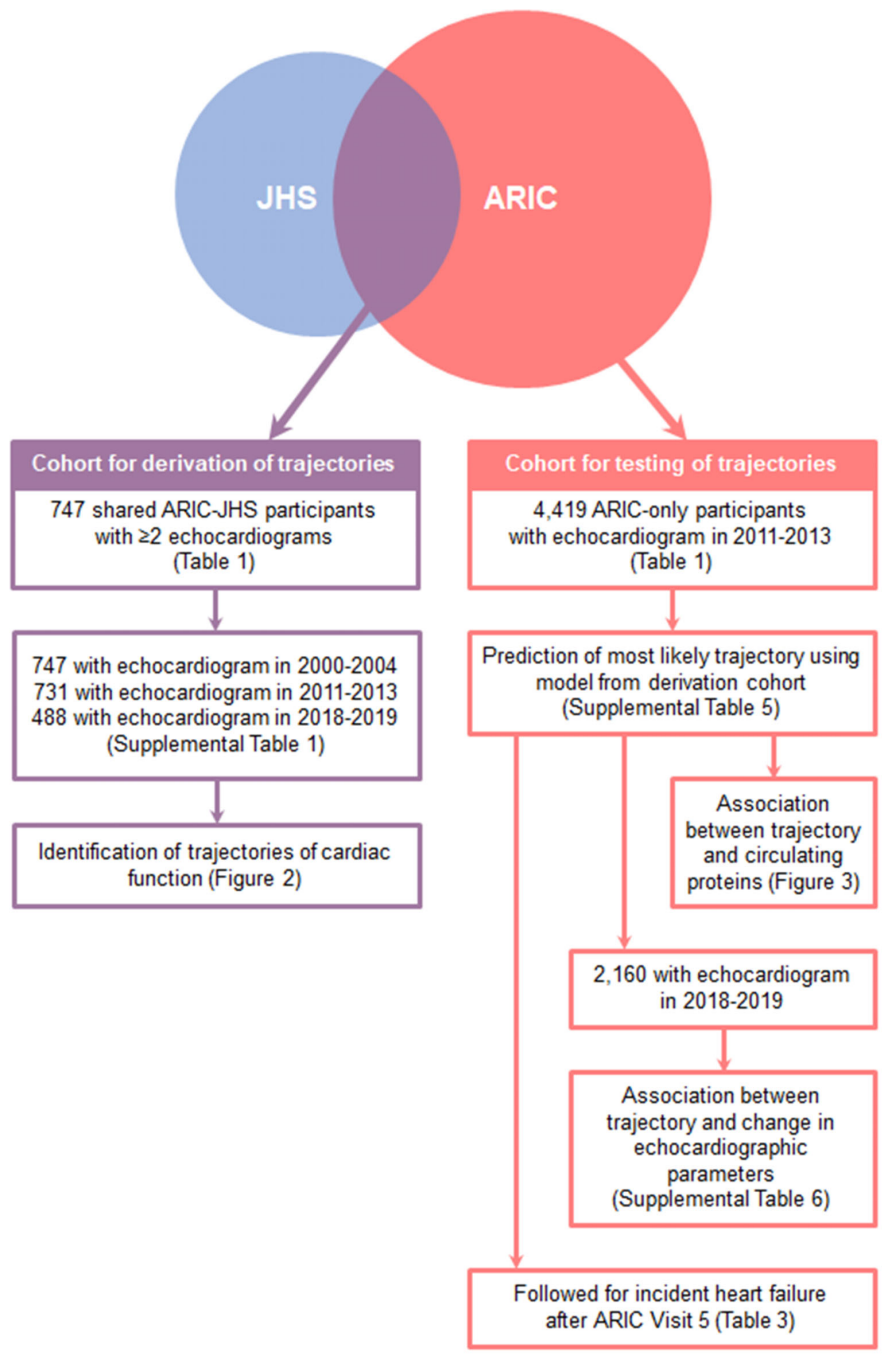
Overview of cohorts used for derivation and testing of trajectories of cardiac function. A Bayesian non‐parametric trajectory approach was used to jointly model longitudinal measures of left ventricular ejection fraction and E/A ratio among 747 shared Atherosclerosis Risk in Communities (ARIC) and Jackson Heart Study (JHS) participants who underwent ≥2 echocardiograms in 2000–2004, 2011–2013, and 2018–2019. Using the resulting model, we predicted the most likely trajectory for 4419 distinct ARIC‐only participants based on single timepoint measures of left ventricular ejection fraction and E/A ratio acquired in 2011–2013. In the testing cohort, we assessed the relationship between predicted trajectory and risk of incident heart failure, changes in echocardiographic parameters and circulating plasma proteins.

Modelling of trajectories of cardiac function was performed in a derivation cohort of 747 shared ARIC‐JHS participants who underwent echocardiography at JHS Visit 1 and ARIC Visit 5 and/or Visit 7 (≥2 echocardiograms). Application of models to predict trajectory membership and test trajectory associations with incident HF and circulating proteins was then performed among 4419 distinct ARIC‐only participants who underwent echocardiography at ARIC Visit 5 (testing cohort). There was no overlap between participants included in each analysis set (*Figure* *1*). We excluded participants with prevalent HF at baseline (JHS Visit 1 for the derivation cohort and ARIC Visit 5 for the testing cohort).

ARIC is an ongoing prospective cohort study of individuals enrolled from four communities in the United States whose design has previously been described in detail^6^ (online supplementary *Methods*). The study protocol was approved by institutional review boards at each field centre and all participants provided written informed consent. JHS is also an ongoing prospective cohort study^7^ that recruited Black American adults from the Jackson metropolitan area between 2000 and 2004 (Visit 1), a subset of whom were also ARIC participants. The JHS was approved by the institutional review boards of Jackson State University, Tougaloo College, and the University of Mississippi Medical Center in Jackson, Mississippi. All study participants provided written informed consent.

### Clinical covariates and incident heart failure

Please refer to online supplementary *Methods* for details regarding assessment of clinical covariates including hypertension, diabetes, chronic kidney disease, coronary heart disease, and atrial fibrillation. Incident HF was based on ARIC and JHS surveillance of hospital discharge codes for study participants and subsequent physician adjudication of medical records from hospitalizations with a potential HF‐related International Classification of Diseases (ICD)‐9 or ICD‐10 code as previously described.^8^, ^9^ In ARIC, incident HFpEF and HFrEF was based on additional abstraction of LVEF from the time of hospitalization^8^ (online supplementary *Methods*).

### Echocardiography

Please refer to online supplementary *Methods* for details regarding echocardiography at JHS Visit 1 and ARIC Visits 5 and 7, which have previously been described in detail.^10^, ^11^ For the purposes of this analysis, for participants in the shared ARIC‐JHS sample, LVEF was calculated using the Teichholz formula^12^ at each visit (JHS Visit 1, ARIC Visit 5, ARIC Visit 7). Key echocardiographic measures (LVEF, E/A ratio) from JHS Visit 1 were then recalibrated against ARIC Visit 5 measures (online supplementary *Methods*).

### Aptamer‐based proteomics

Protein measurements were performed using a multiplexed Slow Off‐rate Modified Aptamer (SOMAmer) assay (SOMAScan v4) as previously described.^13^ After quality control (online supplementary *Methods*), relative protein concentration was available for a total of 4877 aptamers measuring 4697 unique proteins.

### Statistical analysis

#### Bayesian non‐parametric trajectory model

Among participants in the derivation cohort, we applied a Bayesian non‐parametric group‐based trajectory modelling approach^4^ to identify distinct longitudinal progression patterns in LVEF and E/A ratio with age as detailed in online supplementary *Methods*. Both target variables were considered simultaneously by the algorithm. As trajectories are determined based on group‐wise relationships between the predictors and target variables and do not directly represent individual progression, longitudinal data are not required to assign an individual to their most probable trajectory. Thus, we were able to use the derived model and apply Bayes rule to compute the posterior probability of trajectory membership based on single time‐point data to assign participants in the testing cohort to their most probable trajectory group for further analysis.

#### Association between trajectories and outcomes

In the derivation cohort of shared ARIC‐JHS participants, the association between assigned trajectory and risk of incident HF or death post‐Visit 1 was assessed using multivariable Cox proportional hazard models adjusted for age and sex (Model 1) and then further adjusted for obesity, hypertension, diabetes, chronic kidney disease, coronary heart disease (Model 2). In the subsequent analysis of ARIC‐only participants in the testing cohort, we used similar Cox proportional hazard models with additional adjustment for atrial fibrillation to assess associations of assigned trajectory with risk of incident HFpEF or of incident HFrEF. In models with incident HFpEF as the outcome, participants with incident HFrEF or HF with unknown LVEF were censored at the time of that event, and vice versa for analyses with incident HFrEF as the outcome.

#### Identification of proteins associated with trajectories

In the ARIC‐only testing cohort, multinominal logistic regression analyses were conducted to determine if plasma proteins were cross‐sectionally associated with predicted trajectory membership at ARIC Visit 5. For this analysis, the pink trajectory was used as reference. Models were adjusted for age and gender at ARIC Visit 5. Protein levels and age were all centred and scaled to a mean of 0 and standard deviation of 1 to allow for comparison between the different protein models. Multiple testing correction was conducted using the false discovery rate (FDR) method. All analyses were conducted using R (v4.2.0).

#### Mendelian randomization analysis

We applied a two‐sample MR approach^14^, ^15^ to assess potential causal relationships between proteins that were significantly associated with predicted trajectory membership and cardiac structure and function outcomes using summary statistics from large genome‐wide association studies (GWAS) as detailed in online supplementary *Methods*. All the analyses were performed using the R package ‘TwoSampleMR’ (version 0.5.6).^16^

Candidate proteins with MR findings supporting a causal association with cardiac structure/function were assessed as potential drug targets using the ChEMBL database and the druggable genome.^17^, ^18^ Both the database and the supplementary table of druggable genes were queried based on the gene name of the protein of interest.

## Results

### Characteristics of the derivation cohort

Mean age of the 747 shared ARIC‐JHS participants was 64.7 ± 5.0 years at JHS Visit 1 and 30% were male (*Table* *1*). A total of 731 attended ARIC Visit 5 (mean age 74.7 ± 4.9 years) and 488 attended ARIC Visit 7 (mean age 80.5 ± 4.6 years) (online supplementary *Table Appendix* *S1*). Cardiovascular risk factors were common, and the prevalence generally increased over time except for obesity where the prevalence decreased from 51% at JHS Visit 1 to 41% at ARIC Visit 7. Prevalence of cardiovascular disease such as coronary heart disease also increased from JHS Visit 1 to ARIC Visit 7. Mean LVEF was higher at ARIC Visit 7 than JHS Visit 1, while mean LV end‐diastolic and end‐systolic dimension, and E/A ratio were lower. Echocardiographic characteristics at JHS Visit 1 were similar when using the crude (non‐recalibrated) echo measures (online supplementary *Table* *S2*).

**Table 1:** Clinical and echocardiographic characteristics of 747 shared Atherosclerosis Risk in Communities (ARIC) and Jackson Heart Study (JHS) participants and 4419 ARIC‐only participants at baseline

Characteristics	ARIC‐JHS (2000–2004)	ARIC‐only (2011–2013)
Demographics		
*n*	747	4419
Age, years, mean ± SD	64.7 ± 5.0	75.3 ± 5.1
Male sex, *n* (%)	224 (30)	1845 (42)
Black, *n* (%)	747 (100)	394 (9)
Clinical covariates		
Current smoking, *n* (%)	60 (8)	266 (6)
Any prior smoking, *n* (%)	239 (32)	2718 (62)
Obesity, *n* (%)	382 (51)	1372 (31)
Hypertension, *n* (%)	533 (71)	3539 (80)
Diabetes, *n* (%)	186 (25)	1555 (35)
Chronic kidney disease, *n* (%)	32 (4)	1134 (26)
Atrial fibrillation, *n* (%)	0 (0)	167 (4)
Coronary heart disease, *n* (%)	50 (7)	547 (13)
BMI, kg/m^2^, mean ± SD	31.2 ± 6.1	28.2 ± 5.3
Systolic BP, mmHg, mean ± SD	131 ± 16	130 ± 18
Diastolic BP, mmHg, mean ± SD	75 ± 8	66 ± 10
Heart rate, bpm, mean ± SD	62 ± 10	62 ± 10
eGFR, ml/min/1.73 m^2^, mean ± SD	88 ± 17	70 ± 16
Echocardiographic findings, mean ± SD		
LV mean wall thickness, cm	1.1 ± 0.2	0.98 ± 0.13
LV relative wall thickness, cm	0.48 ± 0.12	0.42 ± 0.07
LV end‐diastolic dimension, cm	4.4 ± 0.5	4.4 ± 0.5
LV end‐systolic dimension, cm	2.6 ± 0.5	2.6 ± 0.4
LV mass index, g/m^2^	82 ± 23	79 ± 19
LV ejection fraction, %	70.1 ± 13.6	66.0 ± 5.8
E wave, cm/s	71.3 ± 14.0	65.7 ± 17.0
A wave, cm/s	79.1 ± 17.6	80.6 ± 19.1
E/A ratio	0.92 ± 0.25	0.84 ± 0.23
PASP, mmHg	22.3 ± 6.6	27.8 ± 5.3
LA dimension, cm	3.3 ± 0.6	3.5 ± 0.5

BMI, body mass index; BP, blood pressure; eGFR, estimated glomerular filtration rate; LA, left atrial; LV, left ventricular; PASP, pulmonary artery systolic pressure; SD, standard deviation.

### Trajectories of cardiac function in the derivation cohort

**Figure 2:**
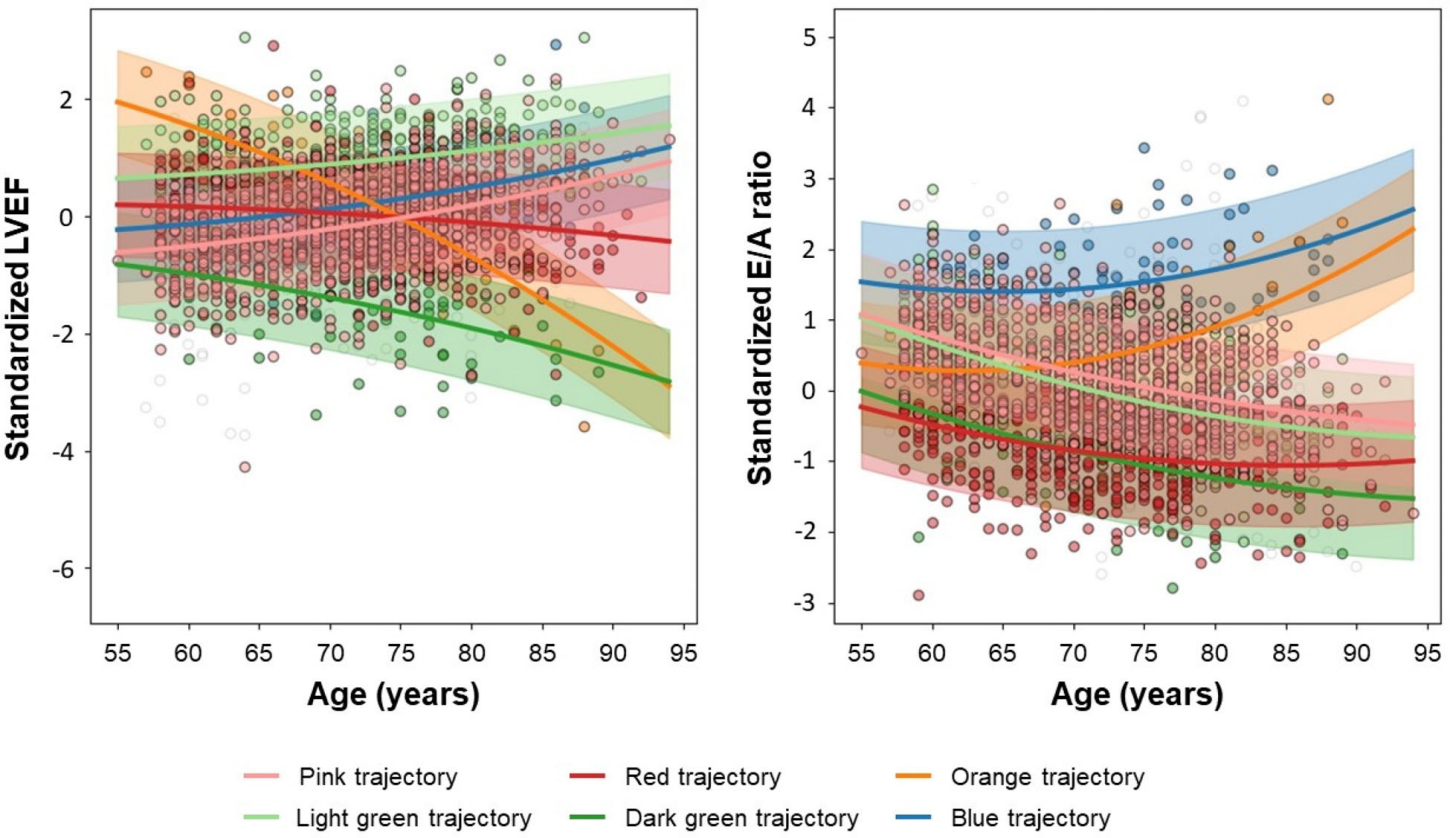
Trajectories of cardiac function (left ventricular ejection fraction [LVEF] and E/A ratio) in 747 shared Atherosclerosis Risk in Communities (ARIC) and Jackson Heart Study (JHS) participants with serial echocardiograms. Among 747 shared ARIC‐JHS participants who underwent ≥2 echocardiograms at JHS Visit 1 (2000–2004), ARIC Visit 5 (2011–2013), and ARIC Visit 7 (2018–2019), we identified six trajectories of cardiac function based on LVEF, E/A ratio and age using a Bayesian non‐parametric trajectory mixture modelling approach.

Using a Bayesian non‐parametric trajectory modelling approach, we identified six trajectories in the derivation cohort (*Figure* *2*): the pink (*n* = 375, 50%) and light green (*n* = 130, 17%) trajectories showed increasing LVEF and decreasing E/A ratio with increasing age with the light green trajectory starting at a higher LVEF. The red trajectory (*n* = 168, 22%) was characterized by a lack of increase in LVEF. Three less common trajectories were also identified. The dark green trajectory (*n* = 29, 4%) showed progressive LVEF decline, the orange trajectory (*n* = 17, 2%) demonstrated a steep LVEF decline and rise in E/A ratio, and the blue trajectory (*n* = 28, 4%) was characterized by rising E/A ratio with age despite increase in LVEF. Similar trajectories were observed in a sensitivity analysis using only individuals with echocardiographic data at all three time points (*n* = 369) (online supplementary *Figure Appendix* *S1*). The age and prevalence of most cardiovascular risk factors were similar across trajectories, although the dark green trajectory had the highest prevalence of men and prevalent coronary heart disease while the red and orange trajectories had the highest prevalence of hypertension (*Table* *2*). Compared to participants in the pink trajectory, at the baseline assessment, those in the red trajectory had higher LV mass index (LVMi), lower E/A ratio, and higher LVEF. Those in the dark green trajectory also had higher LVMi and lower E/A ratio, but lower LVEF. The orange trajectory demonstrated higher LVEF and pulmonary artery systolic pressure while echocardiographic features of the blue trajectory were generally similar to the pink trajectory except E/A ratio was higher. Clinical and echocardiographic characteristics of participants at all three visits according to trajectory are shown in online supplementary *Table* *S3*.

**Table 2:** Clinical and echocardiographic characteristics of 747 shared Atherosclerosis Risk in Communities and Jackson Heart Study participants at the baseline visit (2000–2004) according to trajectory of cardiac function at baseline

	Pink	Light green	Red	Dark green	Orange	Blue	*p*‐value
Demographics							
*n* (%)	375 (50)	130 (17)	168 (22)	29 (4)	17 (2)	28 (4)	
Age, years, mean ± SD	64.5 ± 4.9	64.2 ± 4.8	65.1 ± 5.1	65.6 ± 5.2	65.9 ± 5.3	66.5 ± 4.6*	0.13
Male sex, *n* (%)	124 (33)	14 (11)*	48 (29)	20 (69)*	9 (53)	9 (32)	< 0.001
Clinical covariates							
Current smoking, *n* (%)	35 (9)	7 (6)	12 (7)	2 (7)	3 (20)	1 (4)	0.32
Any prior smoking, *n* (%)	126 (34)	37 (29)	49 (29)	9 (31)	8 (50)	10 (36)	0.50
Obesity, *n* (%)	191 (51)	66 (51)	87 (52)	16 (55)	7 (41)	15 (54)	0.96
Hypertension, *n* (%)	253 (68)	83 (64)	139 (83)*	22 (76)	16 (94)*	20 (71)	< 0.001
Diabetes, *n* (%)	81 (22)	29 (23)	52 (31)*	9 (32)	6 (35)	9 (32)	0.14
Chronic kidney disease, *n* (%)	13 (4)	7 (6)	7 (4)	1 (3)	1 (6)	3 (11)	0.56
Coronary heart disease, *n* (%)	23 (6)	5 (4)	13 (8)	5 (17)*	0 (0)	4 (14)	0.049
BMI, kg/m^2^, mean ± SD	31.3 ± 6.5	31.3 ± 5.9	31.3 ± 5.5	30.8 ± 6.6	30.8 ± 6.2	30.7 ± 5.3	0.99
Systolic BP, mmHg, mean ± SD	130 ± 16	129 ± 16	134 ± 15*	133 ± 14	137 ± 18	127 ± 14	0.02
Diastolic BP, mmHg, mean ± SD	75 ± 8	72 ± 8*	76 ± 9	76 ± 10	75 ± 5	73 ± 8	0.001
Heart rate, bpm, mean ± SD	62 ± 9	61 ± 9	67 ± 10*	65 ± 13	59 ± 10	58 ± 10	<0.001
eGFR, ml/min/1.73 m^2^, mean ± SD	89 ± 16	88 ± 17	88 ± 16	85 ± 15	84 ± 20	84 ± 18	0.33
Echocardiographic findings, mean ± SD							
LV mean wall thickness, cm	1.0 ± 0.2	1.0 ± 0.2	1.1 ± 0.2*	1.1 ± 0.2*	1.1 ± 0.2	1.0 ± 0.2	<0.001
LV relative wall thickness, cm	0.47 ± 0.12	0.47 ± 0.12	0.51 ± 0.11*	0.48 ± 0.12	0.47 ± 0.13	0.45 ± 0.10	0.01
LV end‐diastolic dimension, cm	4.4 ± 0.5	4.3 ± 0.4	4.3 ± 0.5	4.5 ± 0.5	4.5 ± 0.4	4.4 ± 0.4	0.21
LV end‐systolic dimension, cm	2.7 ± 0.4	2.3 ± 0.3*	2.5 ± 0.5*	3.2 ± 0.5 *	2.2 ± 0.4*	2.6 ± 0.5	<0.001
LV mass index, g/m^2^	80 ± 21	80 ± 19	86 ± 26*	92 ± 28 *	90 ± 19	79 ± 23	0.008
LV ejection fraction, %	66.3 ± 15.5	79.6 ± 5.9*	72.3 ± 8.5*	56.8 ± 10.5 *	82.8 ± 7.1*	71.5 ± 8.9	<0.001
E wave, cm/s	74.2 ± 13.8	74.3 ± 12.5	63.1 ± 11.3*	60.5 ± 10.1*	69.2 ± 15.5	78.8 ± 14.7	<0.001
A wave, cm/s	75.5 ± 16.9	79.9 ± 17.2*	88.4 ± 16.5*	80.8 ± 14.6	75.6 ± 18.3	69.1 ± 16.1	<0.001
E/A ratio	1.01 ± 0.21	0.96 ± 0.28	0.69 ± 0.13*	0.72 ± 0.16*	0.93 ± 0.29	1.19 ± 0.24*	<0.001
PASP, mmHg	21.9 ± 6.5	23.3 ± 7.2	21.7 ± 6.3	20.8 ± 5.5	26.4 ± 5.3*	24.0 ± 6.8	0.045
LA dimension, cm	3.3 ± 0.6	3.2 ± 0.5	3.3 ± 0.6	3.2 ± 0.6	3.2 ± 0.8	3.4 ± 0.6	0.73

BMI, body mass index; BP, blood pressure; eGFR, estimated glomerular filtration rate; LA, left atrial; LV, left ventricular; PASP, pulmonary artery systolic pressure; SD, standard deviation.

* *P*<0.05 for comparison with pink trajectory.

Follow‐up was 12 years in the derivation cohort during which time there were 60 incident HF events. Compared to the pink trajectory, a higher risk of incident HF was associated with predicted membership to the red (hazard ratio 1.97, 95% confidence interval 1.04–3.73, *p* = 0.04), dark green (2.78 [1.02–7.56], *p* = 0.045), orange (4.21 [1.42–12.4], *p* = 0.009), and blue (3.86 [1.55–9.63], *p* = 0.004) trajectories. These associations persisted after adjusting for cardiovascular risk factors except for the dark green trajectory for which the association was no longer statistically significant (online supplementary *Table* *S4*). In these models, the orange and blue trajectories were associated with the greatest magnitude of increase in risk, while the red trajectory was associated with an intermediate magnitude of increase in risk.

### Application of trajectories in the testing cohort

We used the Bayesian non‐parametric trajectory model derived in the shared ARIC‐JHS sample to predict the most likely trajectory membership among 4419 distinct ARIC‐only participants based on echocardiography at ARIC Visit 5. In this testing cohort, 2201 (50%) were assigned to the pink trajectory, 676 (15%) were assigned to light green, 942 (21%) to red, 162 (4%) to dark green, 90 (2%) to orange, and 348 (8%) were assigned to the blue trajectory (online supplementary *Table* *S5*). Consistent with trajectories identified in the derivation cohort, ARIC‐only participants assigned to the dark green and orange trajectories demonstrated greater declines in LVEF and global longitudinal strain and increases in LV end‐diastolic volume over 6.6 ± 0.8 years from ARIC Visit 5 to ARIC Visit 7 compared to those assigned to the pink trajectory (online supplementary *Table* *S6*). Similarly, ARIC‐only participants assigned to the orange and blue trajectories demonstrated greater increases in left atrial size, pulmonary artery systolic pressure and E/A ratio between ARIC Visit 5 and Visit 7, while those assigned to the red trajectory demonstrated greater increases in LVESV, compared to those assigned to the pink trajectory, consistent with trajectory patterns identified in the derivation cohort.

Over a median follow‐up of 7.4 years (interquartile range 6.3–7.9 years), there were 379 incident HF events, including 181 HFpEF and 145 HFrEF events, among the testing cohort. In multivariable Cox proportional hazards models adjusted for age, sex, obesity, hypertension, diabetes, chronic kidney disease, coronary heart disease and atrial fibrillation, dark green, orange, and blue trajectories were associated with a heightened risk of HF compared to the pink trajectory (*Table* *3*). While the orange trajectory was associated with heightened risk of both incident HFpEF and HFrEF, the red and dark green trajectories were significantly associated with risk of incident HFrEF but not HFpEF while the blue trajectory was associated with risk of incident HFpEF but not HFrEF. Similar results were observed in sensitivity analysis using trajectory models derived only from ARIC‐JHS participants with three serial echocardiograms (online supplementary *Table* *S7*). Addition of predicted trajectory membership to Cox models with demographics, risk factors, LVEF and E/A ratio significantly improved risk prediction for incident HF (C‐statistic 0.72 vs. 0.73, *p* = 0.01), and incident HFpEF in particular (C‐statistic 0.69 vs. 0.71, *p* = 0.02), based on improvement in the model C‐statistic (online supplementary *Table* *S8*). However, when N‐terminal pro‐B‐type natriuretic peptide (NT‐proBNP) was included in the model, the addition of predicted trajectory membership did not significantly improve risk prediction (online supplementary *Table* *S9*).

**Table 3:** Association between assigned trajectory and risk of incident heart failure over a median follow‐up of 7.4 years in 4419 Atherosclerosis Risk in Communities (ARIC)‐only participants

Trajectory	Prevalence, *n* (%)	Any heart failure			HFpEF			HFrEF		
		Events	Hazard ratio (95% CI)	*p*‐value	Events	Hazard ratio (95% CI)	*p*‐value	Events	Hazard ratio (95% CI)	*p*‐value
Pink	2201 (50)	163	reference		87	reference		50	reference	
Light green	676 (15)	31	0.65 (0.44–0.97)	0.04	16	0.57 (0.32–1.01)	0.05	8	0.59 (0.28–1.26)	0.17
Red	942 (21)	87	1.18 (0.90–1.54)	0.23	37	0.97 (0.66–1.43)	0.87	39	1.67 (1.08–2.57)	0.02
Dark green	162 (4)	38	3.26 (2.27–4.68)	<0.001	7	1.17 (0.54–2.54)	0.70	29	8.08 (5.03–13.0)	<0.001
Orange	90 (2)	21	2.32 (1.44–3.74)	0.001	11	2.33 (1.18–4.58)	0.01	10	3.74 (1.85–7.55)	<0.001
Blue	348 (8)	39	1.64 (1.14–2.35)	0.007	23	1.76 (1.10–2.83)	0.02	9	1.34 (0.65–2.74)	0.42

CI, confidence interval; HF, heart failure; HFpEF, heart failure with preserved ejection fraction; HFrEF, heart failure with reduced ejection fraction; LVEF, left ventricular ejection fraction.

Hazard ratios from Cox proportional hazard models were adjusted for age, sex, obesity, hypertension, diabetes, chronic kidney disease, coronary heart disease and atrial fibrillation. A total of 26 ARIC participants did not have follow‐up data and were excluded from this analysis. Participants with a HF events with LVEF <50% or unknown LVEF at the time of HF hospitalization were censored in analyses with incident HFpEF as endpoint. Conversely, HF events with LVEF ≥50% or unknown LVEF at the time of HF hospitalization were censored in analyses with incident HFrEF as endpoint.

### Identification of proteins associated with trajectories

**Figure 3:**
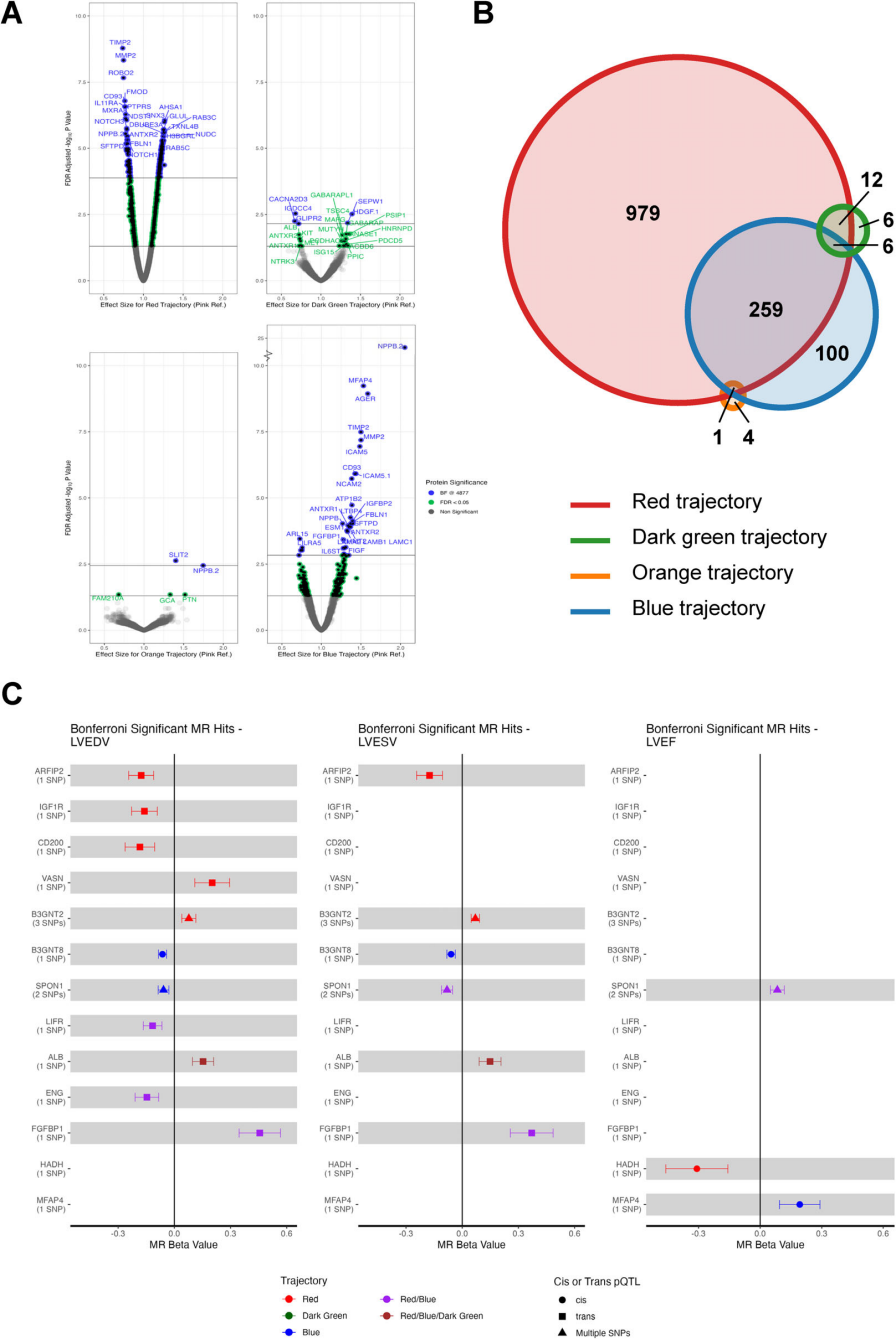
Associations of plasma proteins associated with predicted trajectories membership. (*A*) Volcano plots showing proteins associated with red, dark green, orange, and blue trajectory relative to pink. (*B*) Number of unique and shared proteins associated with each trajectory relative to pink. (*C*) Mendelian randomization (MR) associations of trajectory‐associated proteins with left ventricular structure and function. BF, Bonferroni; FDR, false discovery rate; LVEDV, left ventricular end‐diastolic volume; LVEF, left ventricular ejection fraction; LVESV, left ventricular end‐systolic volume; pQTL, protein quantitative trait loci; SNP, single nucleotide polymorphism.

To explore potential biologic underpinnings of these trajectories of cardiac function, we related 4697 plasma proteins to each high‐risk trajectory (red, dark green, orange, blue) compared to the pink trajectory. Using multinominal logistic regression adjusted for age and sex, 1257 proteins were associated with the red trajectory at FDR <0.05, 366 with the blue trajectory, 24 with the dark green trajectory, and 5 with the orange trajectory (*Figure* *3A*). We identified proteins uniquely associated with each high‐risk trajectory but also proteins shared between several of these trajectories (*Figure* *3B*). Specifically, while a large number of proteins were uniquely associated with the red trajectory (*n* = 979), 100 were uniquely associated with the blue trajectory, 6 with the dark green trajectory, and 4 with the orange trajectory.

Two‐sample MR analyses identified potential causal effects of 13 trajectory‐associated proteins on measures of LV size and LVEF, including six proteins associated with the red trajectory, two proteins associated with the blue trajectory, and four proteins associated with both the red and blue trajectories (*Figure* *3C*, online supplementary *Figure* *S2*). The two proteins uniquely associated with the blue trajectory (UDP‐GlcNAc:betaGal beta‐1,3‐N‐acetylglucosaminyltransferase 2 [B3GNT2], microfibril‐associated glycoprotein 4 [MFAP4]) both had *cis* protein quantitative trait loci (pQTLs) and demonstrated genetic associations with smaller LV size and higher LVEF, consistent with the observed association of the blue trajectory with HFpEF. For the one protein uniquely associated with the red trajectory with a *cis* pQTL (hydroxyacyl‐coenzyme A dehydrogenase [HADH]), genetically higher levels were associated with lower LVEF, consistent with the observed association of the red trajectory with HFrEF. In contrast, the majority of proteins associated with the red trajectory only or with both the red and blue trajectories had *trans* pQTLs and the genetic effects on LV size were variable, suggesting potentially more complicated regulatory effects. Notably, three of these proteins (OX‐2 membrane glycoprotein [CD200], insulin‐like growth factor 1 receptor [IGF1R], endoglin [ENG]) are targeted by existing agents, while five additional proteins – including MFAP4 – are annotated as druggable based on data from the druggable genome database^17^ (online supplementary *Tables* *S10* and *S11*).

## Discussion

Using a novel Bayesian non‐parametric modelling approach, we identified six trajectories of cardiac function based jointly on longitudinal changes in LVEF and E/A ratio in a derivation cohort of 747 Black Americans with ≥2 echocardiograms over an 18‐year period. We then assigned 4419 participants from four communities in the United States to their most likely trajectory based on a single echocardiogram. In this testing cohort, observed changes in echocardiographic measures over time were consistent with predicted trajectory, trajectory membership was differentially associated with risk of incident HFpEF and HFrEF, and information regarding predicted trajectory significantly improved risk prediction for HF and HFpEF beyond demographics, cardiovascular comorbidities, LVEF, and E/A ratio. High‐risk trajectories were associated with several unique and shared proteins with potential causal effects on cardiac structure and function, suggesting potentially distinct underlying molecular pathways (*Graphical Abstract*).

Age‐related changes in cardiac structure and function are well recognized, exacerbated by cardiovascular risk factors, and underlie the age‐associated increase in risk of HF. By analysing average changes in serial echocardiographic measures, previous data from the Framingham Heart Study (FHS) demonstrate that ageing is associated with decreasing LV chamber size, increasing LV wall thickness, and increasing chamber‐level contractility.^2^, ^3^ Furthermore, traditional HF risk factors modify these normal age‐related changes, such that increases in wall thickness and mass are exaggerated, cavity size fails to decrease, and increases in fractional shortening are attenuated. However, while this approach effectively identified a suboptimal pattern of remodelling, HF is heterogeneous with multiple distinct patterns of structural remodelling and functional impairment characterizing HFpEF and HFrEF, and unique antecedent cardiac alterations.

Trajectory modelling using multiple measures of cardiac structure and function jointly holds promise to identify individuals with discrete patterns of cardiac remodelling. Trajectory modelling assumes that there are distinct subpopulations of individuals with distinct progression characteristics and latent risk for adverse events. The goal of trajectory modelling is to identify the number of subgroups, the progression characteristics of each subgroup, and to which subgroup each individual in a data sample is most likely to be a member. Using a Bayesian non‐parametric trajectory modelling approach, we identified six trajectories based jointly on an established measure of systolic function (LVEF) and of diastolic function (E/A ratio). The most common trajectories (pink, light green, red) recapitulated patterns previously identified in the FHS. The pink and light green trajectories were common, associated with the lowest risk of HF, and characterized by increasing LVEF and decreasing E/A ratio with age, consistent with the pattern observed in persons free of HF risk factors in the FHS and concordant with previous cross‐sectional studies of older adults.^19^, ^20^ The red trajectory was the second most common, associated with intermediate risk of HF, and was characterized by a lack of increase in LVEF with age – similar to the pattern observed in persons ageing with cardiovascular risk factors in the FHS.^3^

Novel to this study, we also identified three high‐risk trajectories: dark green characterized by progressive decline in LVEF and an increased risk of incident HFrEF when applied to a separate validation sample; orange characterized by steep LVEF decline and a rise in E/A ratio and increased risk of both HFrEF and HFpEF; and blue characterized by rising E/A ratio despite increase in LVEF (consistent with a primary abnormality of LV stiffness) and an increased risk of incident HFpEF. Importantly, among our testing cohort, the observed changes in cardiac structure and function over 6.6 years in late‐life (from ARIC Visit 5 to 7) were consistent with the expected pattern of change based on predicted trajectory assignment.

We believe there are two major implications of our findings. First, predicted trajectory membership in our testing cohort, based on single timepoint echocardiographic measures, improved risk prediction for incident HF, and specifically HFpEF, beyond demographics, clinical risk factors, and LVEF and E/A ratio. While predicted trajectory did not improve HF risk prediction beyond NT‐proBNP, suggesting this biomarker may be sufficient for predicting HF risk, NT‐proBNP alone is not able to distinguish the underlying cardiac dysfunction (systolic, diastolic, both) or the type of incident HF (HFpEF vs. HFrEF). This distinction is important, as the underlying mechanisms and therefore the optimal preventive therapies likely differ between HF subtypes. These results therefore suggest potential utility of trajectory modelling for risk prediction for incident HFpEF and HFrEF. This is particularly attractive as predicted trajectory can be updated as additional longitudinal data become available. Future studies should evaluate the incremental prognostic value of these trajectories when applied to clinical datasets, and the impact of updating assessments with data from serial studies. If validated in clinical datasets, multiparametric trajectory models could then be used not only to predict risk of HFpEF and HFrEF, but also to identify an individual's cardiac function trajectory early, enabling timely intervention to modify high‐risk trajectories. Emerging therapies such as sodium–glucose co‐transporter 2 inhibitors, glucagon‐like peptide‐1 receptor agonists, and mineralocorticoid receptor antagonists have demonstrated beneficial effects on cardiac structure and function in patients with established HF,^21^, ^22^, ^23^, ^24^ suggesting they may also hold the potential to favourably influence cardiac function trajectories. However, randomized trials are needed to determine whether initiating these agents in individuals on a high‐risk trajectory without overt HF can prevent progression to clinical disease.

Second, proteomic analyses identified several shared and unique circulating proteins differentially associated with the high and intermediate risk trajectories compared with the low‐risk trajectory, a subset of which had potential causal effects on LV structure and function. Importantly, HADH was uniquely associated with the red trajectory, and MR analysis using a *cis* pQTL demonstrated a potential causal association of genetically higher protein levels with lower LVEF – consistent with the observed decline in LVEF with advancing age in this trajectory and its association with risk of HFrEF. HADH is an enzyme involved in β‐oxidation of fatty acids that is enriched in cardiomyocytes,^25^ and possibly involved in endothelial dysfunction in patients with diabetes.^26^ Similarly, both MFAP4 and B3GNT2 were uniquely associated with the blue trajectory, and MR analysis using *cis* pQTLs for each demonstrated potential causal associations of genetically higher protein levels with smaller LV volumes and higher LVEF – consistent with the observed association of this trajectory with risk of HFpEF. While limited data exist regarding the potential role of B3GNT2 in HF development, MFAP4 is a matricellular protein from the fibrinogen‐related protein superfamily with high expression in the heart, lung, and intestine at sites rich in elastic fibres and within blood vessels.^27^ In pre‐clinical studies, MFAP4 has been implicated in remodelling and myocardial fibrosis^28^, ^29^ and MFAP4 levels have previously been associated with risk of cardiovascular diseases relevant to HFpEF pathophysiology, including atrial fibrosis, atrial fibrillation, and pulmonary hypertension.^30^ In contrast, ENG was associated with both the red and blue trajectories, but with higher levels in blue and lower in red relative to the pink trajectory. MR uncovered a potentially causal association of genetically higher ENG with lower LV end‐diastolic volume – consistent with the patterns of remodelling and HF risk observed for the red and blue trajectories. ENG is a co‐receptor for the transforming growth factor‐β family. *ENG* mutations have been causally implicated in hereditary haemorrhagic telangiectasia type 1, pre‐clinical models implicate lack of ENG with eccentric LV remodelling and reduced LVEF,^31^ and anti‐ENG antibody has been tested in clinical trials of cancer treatment.^32^ MR can be used to identify proteins with potential causal roles in HF and may guide therapeutic target discovery,^33^, ^34^ which is of particular interest in HFpEF as prevalence is increasing while effective treatment is limited.^35^ Although our findings do not overlap directly with previously identified GWAS loci, several proteins map to shared biological pathways, such as fibrosis and extracellular matrix remodelling, with prior MR studies supporting causal roles for spondin 1 and MFAP4.^13^, ^34^, ^36^ Together, these findings support biologic underpinnings for the identified trajectories, and suggest potential utility for therapeutic target discovery for interventions to prevent HF.

### Limitations

There were several limitations to the present study. Given the period when JHS Visit 1 echocardiograms were performed, only relatively basic measures of LV systolic and diastolic function were available serially at all three imaging timepoints and therefore used to derive the trajectory model in the shared ARIC‐JHS cohort. As a result, E/e' (a more sensitive measure of diastolic function than E/A) was not available in the derivation cohort due to lack of tissue Doppler imaging at the baseline study. Furthermore, LVEF was calculated by the Teichholz method. Despite this, we were able to show that predicted trajectory membership associated with congruent changes in more contemporary echocardiographic measures of systolic and diastolic function in our testing cohort. More sensitive and comprehensive measures of both systolic and diastolic function are now available, and our findings suggest that future studies incorporating these contemporary measures into model development may enable identification of more refined trajectories and improved risk prediction. Some participants in the shared ARIC‐JHS derivation cohort had differing numbers of available echocardiograms, and the missing echoes may not have been missing at random. However, our findings were consistent when using a model derived only from the subset of participants with echocardiograms available at all three timepoints. The analysis of the association of trajectory with incident HF post‐Visit 1 in JHS (the derivation cohort) is potentially limited by reverse causation as future information (i.e. LVEF and E/A at the second and third echocardiograms) was used to derive trajectory membership. However, this was not an issue in our testing cohort, where echocardiographic data used to assign probably trajectory was acquired prior to follow‐up for HF events. Trajectories were derived in community‐based cohort of Black Americans and applied to a community‐based testing cohort of mainly White Americans, which speaks to generalizability across self‐reported racial groups in the United States. However, the extent to which this model generalizes to other populations and in clinical settings is unclear. MR analyses were based primarily on pQTL and GWAS data from European ancestry populations, which may limit the generalizability of findings to other ancestral groups. Due to differences in the prevalence of predicted trajectories, we had varying power to detect proteomic associations for each trajectory and the difference in the number of identified proteins may simply be a function of different sample sizes between trajectory groups. While the proteomics results support different underlying mechanisms for HF risk in each trajectory, future experimental studies are needed to characterize and test potential mechanisms.

## Conclusions

Using serial echocardiographic data over 18 years in mid‐ to late‐life, multidimensional Bayesian non‐parametric mixture modelling identified high‐risk trajectories of cardiac function with differential associations with risk of HFpEF and HFrEF. Their incremental predictive value highlights the promise of trajectory information to improve clinical HF risk prediction, while proteogenomic analyses indicating partially distinct biologic underpinnings suggest their potential for novel therapeutic target discovery.

### Funding

The Atherosclerosis Risk in Communities (ARIC) study has been funded in whole or in part with federal funds from the National Heart, Lung, and Blood Institute (NHLBI), National Institutes of Health (NIH), Department of Health and Human Services, under Contract nos. (75N92022D00001, 75N92022D00002, 75N92022D00003, 75N92022D00004, 75N92022D00005). The Jackson Heart Study is supported and conducted in collaboration with Jackson State University (HHSN268201800013I), Tougaloo College (HHSN268201800014I), the Mississippi State Department of Health (HHSN268201800015I) and the University of Mississippi Medical Center (HHSN268201800010I, HHSN268201800011I and HHSN268201800012I) contracts from the NHLBI and the National Institute on Minority Health and Health Disparities (NIMHD). SomaLogic Inc. conducted the SomaScan assays in exchange for use of ARIC data. This work was supported in part by NIH/NHLBI grant R01 HL134320. Dr. Shah was supported by NIH/NHLBI grants R01HL135008, R01HL143224, R01HL148218, R01HL150342, R01HL160025, and K24HL152008. Dr. Lutsey was partially supported by K24HL159246.

**Conflict of interest**: T.B.S. reports being a steering Committee member of the Amgen financed GALACTIC‐HF trial and the Boehringer Ingelheim financed SHARP3 trial, being the chief investigator of the Sanofi Pasteur financed NUDGE‐FLU trial, DANFLU‐1 trial, and DANFLU‐2 trial, being a Steering Committee member of ‘LUX‐Dx TRENDS Evaluates Diagnostics Sensors in Heart Failure Patients Receiving Boston Scientific's Investigational ICM System’ trial, attending advisory boards for Sanofi Pasteur, Amgen, CSL Seqirus and GSK, receiving speaker honoraria from Bayer, Novartis, Sanofi Pasteur, GE Healthcare and GSK, received research grants from GE Healthcare, AstraZeneca, Novo Nordisk and Sanofi Pasteur, and having consultant appointments with Novo Nordisk, IQVIA and Parexel. A.M.S. reports consulting fees from Philips Ultrasound and Janssen and research funds from Novartis through Brigham and Women's Hospital. All other authors have nothing to disclose.

## Supporting information


**Appendix S1.** Supporting Information.
