# Supplementary material for: Integrated trajectories of systolic and diastolic function differentially associate with risk for heart failure with preserved and reduced ejection fraction and proteomic profiles
Source: Eur J Heart Fail. 2025 Sep 2;27(12):2906–17. doi: 10.1002/ejhf.70015 (PMC12803532; doi:10.1002/ejhf.70015)
Supplement: Supplementary file 1 — Appendix S1. Supporting Information. [file EJHF-27-2906-s001.docx]

**SUPPLEMENTAL MATERIALS**

**Integrated Trajectories of Systolic and Diastolic Function Differentially Associate with HFpEF and HFrEF and Proteomic Profiles**

Anne Marie Reimer Jensen, MD^a,b,c^; James C. Ross, PhD^d^; Victoria Arthur, PhD^b,e^; Michael E. Hall, MD, MSc^f^; Kunihiro Matsushita, MD, PhD^g^; Brandon Lennep, MD^f^; Pamela L. Lutsey, PhD, MPH^h^; Tor Biering-Sørensen, MD, MSc, PhD, MPH^a,c^; Amil M. Shah, MD, MPH^b,e^

1. Department of Cardiology, Copenhagen University Hospital - Herlev and Gentofte, Gentofte, Denmark
2. Division of Cardiovascular Medicine, Brigham and Women’s Hospital, Boston, MA
3. Center for Translational Cardiology and Pragmatic Randomized Trials, Department of Biomedical Sciences, Faculty of Health and Medical Sciences, University of Copenhagen, Denmark
4. Department of Radiology, Brigham and Women’s Hospital, Boston, MA
5. Division of Cardiology, University of Texas Southwestern Medical Center, Dallas, TX
6. Division of Cardiovascular Diseases, University of Mississippi Medical Center, Jackson, MS
7. Department of Epidemiology, Johns Hopkins Bloomberg School of Public Health, Baltimore, MD
8. Division of Epidemiology & Community Health, University of Minnesota, Minneapolis, MN

**Contents**

- *Supplemental Methods*. Supplemental Methods
- *Supplemental Table 1*. Clinical and echocardiographic characteristics of 747 shared ARIC and JHS participants at all three visits
- *Supplemental Table 2*. Crude and recalibrated echo variables at JHS Visit 1 for shared ARIC-JHS population
- *Supplemental Table 3*. Characteristics of 747 shared ARIC and JHS participants according to trajectory of cardiac function at all three visits
- *Supplemental Table 4*. Association between trajectory of cardiac function and risk of incident heart failure in 747 shared ARIC and JHS participants
- *Supplemental Table 5*. Clinical and echocardiographic characteristics of 4,419 ARIC participants overall and according to trajectory of cardiac function
- *Supplemental Table 6*. Clinical and echocardiographic characteristics of ARIC-only and shared ARIC and JHS participants at ARIC Visit 5
- *Supplemental Table 7*. Association between trajectory and risk of incident heart failure in 4,439 ARIC-participants and according to trajectory derived from participants with 3 echocardiograms
- *Supplemental Table 8*. Incremental predictive value of predicted trajectory membership among ARIC-only participants
- *Supplemental Table 9.* Incremental predictive value of predicted trajectory membership among ARIC-only participants with NT-proBNP
- *Supplemental Table 10*. Drugs and Clinical Candidates from the ChEMBL database
- *Supplemental Table 11.* Trajectory associated proteins annotated as potentially druggable in The Druggable Genome
- *Supplemental Figure 1.* Trajectories of cardiac function (LVEF and E/A-ratio) in 369 shared ARIC-JHS participants with 3 echocardiograms
- *References*

**Supplemental Methods**

*The Atherosclerosis Risk in Communities (ARIC) study*

ARIC is an ongoing prospective cohort study of individuals enrolled from four communities in the United States^1^: Forsyth County, North Carolina; Jackson, Mississippi; suburban Minneapolis, Minnesota; and Washington County, Maryland. Between 1987 and 1989, 15,792 middle-aged individuals attended the first study visit. At the fifth study visit (2011-13), 6,538 of 9,870 surviving participants returned and 6,118 underwent echocardiography. Between 2018 and 2019, 3,408 of 5,058 surviving participants returned for the seventh study visit and 3,046 underwent echocardiography.

*Assessment of clinical covariates*

Prevalent hypertension was defined as systolic blood pressure greater than or equal to 140 mmHg or diastolic blood pressure greater than or equal to 90 mmHg or use of blood pressure-lowering medication. Diabetes mellitus was defined as a hemoglobin A_1c_ ≥6.5%, fasting plasma glucose ≥126 mg/dL, or self-report of diabetes medication use. History of smoking was derived from questionnaire or participant interview. Estimated glomerular filtration rate (eGFR) was calculated based on serum creatinine using the Chronic Kidney Disease Epidemiology equation^2^. Chronic kidney disease was defined as eGFR <60 mL/min/1.73m^2^. Prevalent heart failure (HF) was based on hospitalization with an International Classification of Diseases, ninth revision (ICD-9), discharge code of 428 before 2005^3^ with additional physician adjudication of hospitalizations with HF related ICD-9 or ICD-10 codes since 2005^4^. Coronary heart disease (CHD) was defined as a history of myocardial infarction or coronary intervention (percutaneous coronary intervention, coronary bypass surgery), or (in JHS) electrocardiogram evidence of a prior myocardial infarction or (in ARIC) regional wall motion abnormality on echocardiography. Atrial fibrillation was ascertained based on ECGs and hospital discharge records^5^.

*Ascertainment of incident heart failure events*

Incident HF was based on ARIC and JHS surveillance of hospital discharge codes for study participants and subsequent physician adjudication of medical records from hospitalizations with a potential HF-related ICD-9 or ICD-10 code as previously described^4,6^. For analysis of shared ARIC-JHS participants, follow-up was from JHS adjudication after JHS Visit 1 through December 31, 2016. In ARIC-only analysis, HF hospitalizations were categorized as HFpEF if LVEF abstracted from medical records at the time of hospitalization was 50% or greater and HFrEF if the abstracted LVEF was less than 50%^7^. Of 379 incident HF events after ARIC Visit 5 through December 31, 2019, information on LVEF at HF diagnosis was available in 326 (86%); 145 with HFrEF and 181 with HFpEF. Deaths were ascertained by ARIC surveillance and linkage to the National Death Index^8^.

*Echocardiography*

In JHS Visit 1, echocardiograms were recorded by trained sonographers and interpreted by experienced cardiologists in the Echocardiography Reading Center at the University of Mississippi Medical Center (Jackson, MS)^9^. LVEF was calculated from LV end-diastolic (LVEDD) and end-systolic (LVESD) dimension using Teichholz method^10^: ((7/(2.4+LVEDD)*(LVEDD)3)- (7/(2.4+ LVESD)*(LVESD)3))/ (7/(2.4+ LVEDD)* (LVEDD)3)*100%. When a quantitative LVEF was not available, semiquantitative measures were used (LVEF estimated to nearest 5%). The JHS Echocardiography Manual is available on the JHS website (https://www.jacksonheartstudy.org/Portals/0/pdf/manuals1/Echocardiography_manual6.pdf).

In ARIC, echocardiograms were performed by certified study sonographers at all four field centers using uniform imaging equipment and following a standardized image acquisition protocol at both visits as previously described^11^. Quantitative measures were performed by blinded analysts at a dedicated Echocardiography Reading Center at Brigham and Women’s Hospital (Boston, MA), in a manner consistent with the recommendation of the American Society of Echocardiography^12^. The LVEF was derived from LV volumes calculated by the modified Simpson method using the apical 4- and 2-chamber views. In both studies, E/A ratio was calculated based on the peak early and late diastolic transmitral flow assessed at the level of the mitral leaflet tips by pulse wave Doppler in the apical 4-chamber view.

*Recalibration of echo measures in the Jackson Heart Study (JHS)*

Key echocardiographic measures (LVEF, E/A ratio) from JHS Visit 1 were recalibrated against ARIC Visit 5 measures based on re-analysis of 400 JHS Visit 1 studies at the ARIC Echocardiography Reading Center at Brigham and Women’s Hospital. These studies were selected to be representative of the full JHS Visit 1 echocardiography sample with respect to age, gender, and key cardiovascular risk factors. For each echocardiographic measure, studies with differences between the original and remeasured values that were >3 standard deviations from the mean difference were removed in an iterative outlier removal process consistent with recalibration approaches previously applied to serial laboratory measures^13^. Means and standard deviations of differences were recalculated, and the process was repeated until no outliers remained. Recalibration equations were then derived from Deming regression coefficients from regression of the original versus re-measured values.

*Aptamer-based Proteomics*

Blood plasma collected using standard protocols at ARIC sites at Visit 5 were stored at -80ºC and sent to SomaLogic for quantification. The relative concentration levels of plasma proteins were determined using the SOMAmer assay, which utilizes small pieces of single strand DNA with modified aptamer regions designed to bind to proteins based on a particular sequence or three-dimensional structure. DNA detection technology is then used to identify and quantify the proteins. Additional details on the assay and its performance have been previously published^14^.

ARIC data sets were combined with data from healthy controls and then normalized. Samples were flagged if at least one of the four sample calibration factors was outside of the acceptance criteria range of 0.4-2.5. These factors include hybridization control normalization factor, normalization factor for a dilution factor of 0.005%, normalization factor for a dilution factor of 0.5% and normalization factor for a dilution factor of 20%. SOMAmers were flagged if at least one intraplate calibration factor was out of the acceptance criteria of 0.8-1.2. Additional quality control procedures were implemented by the ARIC study. SOMAmer measures were log2 transformed to correct for skewness in the data distribution. Blind duplicates were run for 4% of participants (185 of 5327 individuals at Visit 5). The median inter-assay coefficient of variation for SOMAmers calculated using Bland-Altman analysis was 4.67%. Quality control outliers were excluded as described below. The median split sample reliability coefficient was 0.94. Additionally, manual annotation was completed for six UniProt IDs and three protein names. Of the 5,284 SOMAmers available before quality control exclusion, 93 aptamers were excluded from analysis as they had an inter-assay coefficient of variation > 50%. Additionally, 12 proteins with variance < 0.01 on a log-scale were excluded. SOMAmers that bound to Fc mouse (228), contaminants (15), or non-proteins (70) including hybridization control elution, non-human proteins, non-biotin, non-cleavable, or spuriomer molecules were also excluded. Samples deemed to be outliers for each SOMAmer, defined as values outside of 5 times the standard deviation of the log2 scaled same mean, were winsorized.

*Bayesian non-parametric trajectory model*

Among participants in the derivation cohort, we applied a Bayesian nonparametric group-based trajectory modeling approach^15^ to identify distinct longitudinal progression patterns in LVEF and E/A ratio with age. Both target variables were considered simultaneously by the algorithm. Target variable residuals are assumed to be normally distributed within each trajectory. Therefore, we transformed LVEF and E/A ratio measures prior to trajectory modeling. We treated LVEF as compositional data and applied the isometric log-ratio transformation to these measures, and we log-transformed the E/A ratio values. The predictors used in the trajectory model included age centered, (age centered)^2^, and an intercept term, where age centered is the age variable minus 50. In a sensitivity analysis, we restricted trajectory modeling to participants with three longitudinal measurements (JHS Visit 1, ARIC Visit 5, and ARIC Visit 7). Trajectory modeling proceeded by performing Bayesian approximate inference with 100 random initializations, using 200 iterations per optimization run. The trajectory model with the highest WAIC2 score^16^ (an information criteria measure that captures goodness of fit while balancing model complexity) was used for subsequent analysis.

*Two-sample Mendelian randomization analysis*

We applied a two-sample Mendelian randomization (MR) approach^17,18^ to assess potential causal relationships between proteins that were significantly associated with predicted trajectory membership and cardiac structure and function outcomes. To minimize bias, each analysis used summary statistics from two independent European samples for exposure and outcome data, respectively. Instrumental variables (IVs) for protein quantitative trait loci (pQTLs) were obtained from the Fenland study^19^ (N=10,708) which included summary statistics for 4,775 proteins measured by SOMAscan; the AGES cohort study^20^ (N=5,368) with summary statistics for 4,782 SOMAscan proteins; the INTERVAL study^21^ (N=3,301) with summary statistics for 2,994 proteins measured with SOMAscan; the KORA study^22^ (N=1,000) with summary statistics for 1,124 SOMAscan proteins; a GWAS meta-analysis^23^ (N=8,293) of three independent Finnish cohorts with summary statistics for 41 cytokines measured using Bio-Rad’s premixed Bio-Plex Pro Human Cytokine Assay; the UK Household Longitudinal Study (UKHLS)^24^ (N=9,961) with summary statistics for serum albumin levels measured in plasma; the MANOLIS^25^ study (N=1,328) with summary statistics for 257 proteins from three Olink panels (CVDII, CVDIII, and Metabolism); and the SCALLOP consortium^26^ (N=30,931 across 14 studies) with summary statistics for 90 proteins measured using the Olink CVD-I panel.

Summary statistics for cardiac structure and function outcomes (LV end-diastolic volume (LVEDV), LV end-systolic volume (LVESV) and LVEF) were obtained from the UK Biobank^27^ (N=36,041). IVs were selected if they reached genome-wide significance (*p*<5 x 10^-8^), and the selected IVs were further clumped with r^2^<0.001. Wald tests or inverse variance weighted tests, for more than one IV, were performed to calculate a causal estimate. Cochran's heterogeneity test was used to test the heterogeneity if more than two IVs were included. The MR Egger^28^ method was used to test for the presence of horizontal pleiotropy if three or more IVs were included. The significance thresholds for MR association were determined using Bonferroni correction for the number of independent sets of IVs tested, *p*<1.14x10^-04^ for the red trajectory (0.05/437), *p*<3.85x10^-03^ for the dark green trajectory (0.05/13), *p*<0.0125 for the orange trajectory (0.05/4), and *p*<4x10^-04^ for the blue trajectory (0.05/125). For the observed significant associations, we further conducted a backward MR with IVs for cardiac structure and function as exposure and IVs for proteins as outcome, to detect potential reverse causality. All the analyses were performed using the R package “TwoSampleMR” (version 0.5.6)^29^.

**Supplemental Table 1.** Clinical and echocardiographic characteristics of 747 shared ARIC and JHS participants at all three visits

| **Characteristics** | **1^st^ Echocardiogram**  **(2000-04)** | **2^nd^ Echocardiogram**  **(2011-13)** | **3^rd^ Echocardiogram**  **(2018-19)** |
| --- | --- | --- | --- |
| *Demographics* | | | |
| N | 747 | 731 | 488 |
| Age, mean ± SD, years | 64.7 ± 5.0 | 74.7 ± 4.9 | 80.5 ± 4.6 |
| Male sex, n (%) | 224 (30) | 220 (30) | 139 (28) |
| *Clinical covariates* | | | |
| Current smoking, n (%) | 60 (8) | 35 (55) | 32 (7) |
| Any prior smoking, n (%) | 239 (32) | 358 (52) | 279 (63) |
| Obesity, n (%) | 382 (51) | 352 (48) | 196 (41) |
| Hypertension, n (%) | 533 (71) | 689 (94) | 719 (97) |
| Diabetes, n (%) | 186 (25) | 335 (46) | 354 (62) |
| Chronic kidney disease, n (%) | 32 (4) | 186 (26) | 144 (33) |
| Atrial fibrillation, n (%) | 0 (0) | 25 (4) | 64 (9) |
| Coronary heart disease, n (%) | 50 (7) | 68 (9) | 57 (12) |
| Heart failure, n (%) | 0 (0) | 40 (6) | 74 (10) |
| BMI, mean ± SD | 31.2 ± 6.1 | 30.6 ± 6.1 | 29.6 ± 5.7 |
| Systolic BP, mean ± SD, mmHg | 131 ± 16 | 135 ± 19 | 135 ± 19 |
| Diastolic BP, mean ± SD, mmHg | 75 ± 8 | 70 ± 10 | 65 ± 11 |
| Heart rate, mean ± SD, beats/min | 62 ± 10 | 64 ± 11 | 64 ± 11 |
| eGFR, mean ± SD, ml/min/1.73m^2^ | 88 ± 17 | 73 ± 21 | 68 ± 22 |
| *Echocardiographic findings, mean ± SD* | | | |
| LV mean wall thickness, cm | 1.1 ± 0.2 | 1.0 ± 0.1 | 1.1 ± 0.1 |
| LV relative wall thickness, cm | 0.48 ± 0.12 | 0.45 ± 0.08 | 0.48 ± 0.09 |
| LV end-diastolic dimension, cm | 4.4 ± 0.5 | 4.3 ± 0.5 | 4.2 ± 0.5 |
| LV end-systolic dimension, cm | 2.6 ± 0.5 | 2.5 ± 0.5 | 2.3 ± 0.5 |
| LV mass index, g/m^2^ | 82 ± 23 | 76 ± 19 | 79 ± 19 |
| LV ejection fraction, % | 70.1 ± 13.6 | 71.1 ± 9.2 | 75.0 ± 10.8 |
| E wave, cm/s | 71.3 ± 14.0 | 65.8 ± 17.5 | 73.3 ± 17.8 |
| A wave, cm/s | 79.1 ± 17.6 | 83.2 ± 18.3 | 92.1 ± 20.4 |
| E/A ratio | 0.92 ± 0.25 | 0.81 ± 0.22 | 0.82 ± 0.25 |
| PASP, mmHg | 22.3 ± 6.6 | 27.9 ± 6.0 | 33.2 ± 8.5 |
| LA dimension, cm | 3.3 ± 0.6 | 3.4 ± 0.5 | 3.3 ± 0.5 |

Abbreviations: ARIC, Atherosclerosis Risk in Communities; BMI, body mass index; BP, blood pressure; body surface area; eGFR, estimated glomerular filtration rate; JHS, Jackson Heart Study; LA, left atrial; LV, left ventricular; PASP, pulmonary artery systolic pressure.

**Supplemental Table 2.** Crude and recalibrated echo variables at JHS Visit 1 for shared ARIC-JHS population

| **Characteristics, mean ± SD** | **Crude** | **Recalibrated** |
| --- | --- | --- |
| LV mean wall thickness, cm | 0.9 ± 0.1 | 1.1 ± 0.2 |
| LV relative wall thickness, cm | 0.37 ± 0.07 | 0.48 ± 0.12 |
| LV end-diastolic dimension, cm | 4.8 ± 0.4 | 4.4 ± 0.5 |
| LV end-systolic dimension, cm | 2.9 ± 0.4 | 2.6 ± 0.5 |
| LV mass, g | 153 ± 41 | 161 ± 47 |
| LV ejection fraction, % | 70.1 ± 10.4 | 70.1 ± 13.6 |
| E wave, cm/s | 80.6 ± 18.5 | 71.3 ± 14.0 |
| A wave, cm/s | 87.9 ± 18.9 | 79.1 ± 17.6 |
| E/A ratio | 0.94 ± 0.24 | 0.92 ± 0.25 |
| PASP, mmHg | 24.4 ± 7.0 | 22.3 ± 6.6 |
| LA dimension, cm | 3.5 ± 0.4 | 3.3 ± 0.6 |

Echocardiographic measures from JHS Visit 1 was recalibrated against ARIC Visit 5 measures based on re-analysis of 400 representative studies at the ARIC Echocardiography Reading Center at Brigham and Women’s Hospital. For each echocardiographic measure, studies with differences between the original and remeasured values that are >3 standard deviations from the mean difference are removed in an iterative outlier removal process consistent with recalibration approaches previously applied to serial laboratory measures^10^. Means and standard deviations of differences were recalculated, and the process was repeated until no outliers remained. Recalibration equations were then derived from Deming regression coefficients from regression of the original versus re-measured values.

Abbreviations: ARIC, Atherosclerosis Risk in Communities; BMI, body mass index; BP, blood pressure; body surface area; eGFR, estimated glomerular filtration rate; JHS, Jackson Heart Study; LA, left atrial; LV, left ventricular; PASP, pulmonary artery systolic pressure*.*

**Supplemental Table 3.** Characteristics of 747 shared ARIC and JHS participants according to trajectory of cardiac function at all three visits

|  | **Pink** | **Light green** | **Red** | **Dark green** | **Orange** | **Blue** | ***p*-value** |
| --- | --- | --- | --- | --- | --- | --- | --- |
| N, (%)  *- JHS Visit 1*  *- ARIC Visit 5*  *- ARIC Visit 7* | 375  369  241 | 130  126  94 | 168  166  106 | 29  28  17 | 17  16  10 | 28  26  20 | - |
| Age, years  *- JHS Visit 1*  *- ARIC Visit 5*  *- ARIC Visit 7* | 64.5 ± 4.9  74.5 ± 4.8  80.1 ± 4.4 | 64.2 ± 4.8  74.1 ± 4.6  80.4 ± 4.5 | 65.1 ± 5.1  74.9 ± 5.1  80.9 ± 4.8 | 65.6 ± 5.2  75.4 ± 5.2  81.1 ± 5.3 | 65.9 ± 5.3  77.1 ± 4.6  82.4 ± 5.2 | 66.5 ± 4.6  76.3 ± 4.9  82.8 ± 4.0 | 0.13  0.06  0.07 |
| Male, n (%)  *- JHS Visit 1*  *- ARIC Visit 5*  *- ARIC Visit 7* | 124 (33)  122 (33)  78 (32) | 14 (11)  13 (10)  9 (10) | 48 (29)  48 (29)  30 (28) | 20 (69)  20 (71)  11 (65) | 9 (53)  9 (56)  6 (60) | 9 (32)  8 (31)  5 (25) | < 0.001  < 0.001  < 0.001 |
| Current smoking, n (%)  *- JHS Visit 1*  *- ARIC Visit 5*  *- ARIC Visit 7* | 35 (9)  17 (5)  17 (7) | 7 (6)  3 (2)  6 (6) | 12 (7)  12 (7)  7 (7) | 2 (7)  0 (0)  1 (6) | 3 (20)  1 (6)  1 (10) | 1 (4)  2 (8)  0 (0) | 0.32  0.34  0.89 |
| Any prior smoking, n (%)  *- JHS Visit 1*  *- ARIC Visit 5*  *- ARIC Visit 7* | 126 (34)  183 (52)  146 (67) | 37 (29)  58 (50)  48 (56) | 49 (29)  73 (48)  59 (62) | 9 (31)  16 (62)  7 (54) | 8 (50)  12 (86)  8 (89) | 10 (36)  16 (64)  11 (61) | 0.50  0.07  0.26 |
| Obesity, n (%)  *- JHS Visit 1*  *- ARIC Visit 5*  *- ARIC Visit 7* | 191 (51)  183 (50)  104 (44) | 66 (51)  60 (48)  34 (37) | 87 (52)  77 (46)  39 (39) | 16 (55)  14 (50)  7 (41) | 7 (41)  5 (33)  5 (50) | 15 (54)  13 (50)  7 (35) | 0.96  0.86  0.81 |
| Hypertension, n (%)  *- JHS Visit 1*  *- ARIC Visit 5*  *- ARIC Visit 7* | 253 (68)  335 (91)  352 (96) | 83 (64)  123 (98)  128 (98) | 139 (83)  163 (98)  166 (99) | 22 (76)  28 (100)  29 (100) | 16 (94)  16 (100)  17 (100) | 20 (71)  24 (92)  27 (96) | < 0.001  0.002  0.36 |
| Diabetes, n (%)  *- JHS Visit 1*  *- ARIC Visit 5*  *- ARIC Visit 7* | 81 (22)  166 (45)  175 (61) | 29 (23)  45 (36)  48 (46) | 52 (31)  89 (54)  94 (73) | 9 (32)  14 (50)  14 (78) | 6 (35)  9 (56)  9 (75) | 9 (32)  12 (46)  14 (61) | 0.14  0.07  0.001 |
| Chronic kidney disease, n (%)  *- JHS Visit 1*  *- ARIC Visit 5*  *- ARIC Visit 7* | 13 (4)  93 (26)  70 (32) | 7 (6)  27 (22)  22 (26) | 7 (4)  42 (26)  34 (36) | 1 (3)  8 (29)  4 (33) | 1 (6)  8 (50)  8 (80) | 3 (11)  8 (31)  6 (35) | 0.56  0.28  0.03 |

Abbreviations: ARIC, Atherosclerosis Risk in Communities; body surface area; eGFR, estimated glomerular filtration rate; JHS, Jackson Heart Study; LA, left atrial; LV, left ventricular; PASP, pulmonary artery systolic pressure.

**Supplemental Table 3 (continued).** Characteristics of 747 shared ARIC and JHS participants according to trajectory of cardiac function at all three visits

|  | **Pink** | **Light green** | **Red** | **Dark green** | **Orange** | **Blue** | ***p*-value** |
| --- | --- | --- | --- | --- | --- | --- | --- |
| Atrial fibrillation, n (%)  *- JHS Visit 1*  *- ARIC Visit 5*  *- ARIC Visit 7* | .  8 (2)  21 (6) | .  3 (2)  5 (4) | .  8 (5)  25 (15) | .  0 (0)  3 (11) | .  3 (19)  5 (31) | .  3 (12)  5 (19) | .  0.001  < 0.001 |
| Coronary heart disease, n (%)  *- JHS Visit 1*  *- ARIC Visit 5*  *- ARIC Visit 7* | 23 (6)  30 (8)  21 (9) | 5 (4)  9 (7)  9 (10) | 13 (8)  17 (10)  15 (14) | 5 (17)  3 (11)  3 (18) | 0 (0)  3 (19)  4 (40) | 4 (14)  6 (23)  5 (25) | 0.049  0.11  0.01 |
| Heart failure, n (%)  *- JHS Visit 1*  *- ARIC Visit 5*  *- ARIC Visit 7* | .  10 (3)  27 (7) | .  7 (6)  10 (8) | .  13 (8)  21 (13) | .  5 (18)  7 (25) | .  2 (13)  4 (25) | .  3 (12)  5 (19) | .  0.002  0.003 |
| Body mass index, kg/m^2^  *- JHS Visit 1*  *- ARIC Visit 5*  *- ARIC Visit 7* | 31.3 ± 6.5  30.8 ± 6.4  30.1 ± 6.1 | 31.3 ± 5.9  30.7 ± 6.1  29.1 ± 5.4 | 31.3 ± 5.5  30.3 ± 5.5  29.2 ± 5.5 | 30.8 ± 6.6  30.9 ± 6.6  28.9 ± 6.0 | 30.8 ± 6.2  28.6 ± 5.3  28.5 ± 4.9 | 30.7 ± 5.3  29.8 ± 5.5  28.9 ± 4.6 | 0.99  0.75  0.59 |
| Heart rate, bpm  *- JHS Visit 1*  *- ARIC Visit 5*  *- ARIC Visit 7* | 62 ± 9  63 ± 10  63 ± 10 | 61 ± 9  62 ± 10  63 ± 10 | 67 ± 10  68 ± 12  66 ± 9 | 65 ± 13  68 ± 12  68 ± 13 | 59 ± 10  62 ± 12  71 ± 33 | 58 ± 10  58 ± 9  59 ± 11 | < 0.001  < 0.001  0.01 |
| eGFR, mL·min^-1^·1.73m^-2^  *- JHS Visit 1*  *- ARIC Visit 5*  *- ARIC Visit 7* | 89 ± 16  74 ± 20  69 ± 21 | 88 ± 17  76 ± 21  72 ± 19 | 88 ± 16  72 ± 21  65 ± 22 | 85 ± 15  73 ± 23  65 ± 37 | 84 ± 20  64 ± 26  47 ± 17 | 84 ± 18  67 ± 26  65 ± 24 | 0.33  0.12  0.01 |
| LV mean wall thickness, cm  *- JHS Visit 1*  *- ARIC Visit 5*  *- ARIC Visit 7* | 1.04 ± 0.17  0.99 ± 0.13  1.06 ± 0.13 | 1.04 ± 0.17  0.98 ± 0.10  1.05 ± 0.12 | 1.11 ± 0.19  1.03 ± 0.16  1.09 ± 0.17 | 1.13 ± 0.21  0.99 ± 0.11  1.00 ± 0.10 | 1.11 ± 0.17  1.08 ± 0.19  1.08 ± 0.20 | 1.03 ± 0.21  1.01 ± 0.19  1.08 ± 0.23 | < 0.001  0.001  0.25 |
| LV relative wall thickness, cm  *- JHS Visit 1*  *- ARIC Visit 5*  *- ARIC Visit 7* | 0.47 ± 0.12  0.44 ± 0.08  0.48 ± 0.09 | 0.47 ± 0.12  0.43 ± 0.06  0.48 ± 0.08 | 0.51 ± 0.11  0.46 ± 0.09  0.49 ± 0.12 | 0.48 ± 0.12  0.42 ± 0.07  0.42 ± 0.07 | 0.47 ± 0.13  0.50 ± 0.10  0.49 ± 0.08 | 0.45 ± 0.10  0.44 ± 0.10  0.48 ± 0.13 | 0.01  0.02  0.32 |

Abbreviations: ARIC, Atherosclerosis Risk in Communities; body surface area; eGFR, estimated glomerular filtration rate; JHS, Jackson Heart Study; LA, left atrial; LV, left ventricular; PASP, pulmonary artery systolic pressure.

**Supplemental Table 3 (continued).** Characteristics of 747 shared ARIC and JHS participants according to trajectory of cardiac function at all three visits

|  | **Pink** | **Light green** | **Red** | **Dark green** | **Orange** | **Blue** | ***p*-value** |
| --- | --- | --- | --- | --- | --- | --- | --- |
| LV end-diastolic dimension, cm  *- JHS Visit 1*  *- ARIC Visit 5*  *- ARIC Visit 7* | 4.37 ± 0.47  4.26 ± 0.46  4.17 ± 0.49 | 4.32 ± 0.42  4.21 ± 0.41  4.07 ± 0.37 | 4.32 ± 0.48  4.26 ± 0.49  4.09 ± 0.52 | 4.53 ± 0.45  4.58 ± 0.93  4.74 ± 0.70 | 4.46 ± 0.41  4.28 ± 0.55  4.28 ± 0.37 | 4.36 ± 0.44  4.43 ± 0.58  4.28 ± 0.63 | 0.21  0.01  0.002 |
| LV end-systolic dimension, cm  *- JHS Visit 1*  *- ARIC Visit 5*  *- ARIC Visit 7* | 2.73 ± 0.43  2.57 ± 0.39  2.36 ± 0.43 | 2.25 ± 0.32  2.18 ± 0.29  1.96 ± 0.28 | 2.51 ± 0.45  2.57 ± 0.39  2.38 ± 0.42 | 3.15 ± 0.45  3.35 ± 0.89  3.79 ± 0.68 | 2.19 ± 0.41  2.71 ± 0.50  3.14 ± 0.61 | 2.57 ± 0.46  2.49 ± 0.36  2.29 ± 0.63 | < 0.001  < 0.001  < 0.001 |
| LV mass index, g/m^3^  *- JHS Visit 1*  *- ARIC Visit 5*  *- ARIC Visit 7* | 80.4 ± 21.4  73.5 ± 16.6  78.5 ± 19.3 | 80.3 ± 19.1  73.1 ± 14.8  77.9 ± 15.2 | 85.9 ± 25.5  79.0 ± 21.6  79.7 ± 18.2 | 91.6 ± 28.5  81.6 ± 36.7  86.5 ± 27.2 | 90.1 ± 18.6  85.6 ± 25.0  90.8 ± 24.6 | 79.1 ± 22.8  81.8 ± 26.2  81.5 ± 28.9 | 0.008  < 0.001  0.42 |
| LV ejection fraction, %  *- JHS Visit 1*  *- ARIC Visit 5*  *- ARIC Visit 7* | 66.3 ± 15.5  70.0 ± 7.8  74.7 ± 8.6 | 79.6 ± 5.9  79.5 ± 5.3  83.2 ± 4.6 | 72.3 ± 8.5  69.9 ± 8.2  73.0 ± 8.0 | 56.8 ± 10.5  52.5 ± 11.1  41.1 ± 8.6 | 82.8 ± 7.1  67.0 ± 6.7  53.2 ± 16.6 | 71.5 ± 8.9  74.8 ± 5.0  78.0 ± 9.1 | < 0.001  < 0.001  < 0.001 |
| E wave, cm/s  *- JHS Visit 1*  *- ARIC Visit 5*  *- ARIC Visit 7* | 74.2 ± 13.8  67.6 ± 15.4  74.3 ± 16.7 | 74.3 ± 12.5  67.7 ± 16.6  73.4 ± 14.8 | 63.1 ± 11.3  58.5 ± 16.7  66.9 ± 17.6 | 60.5 ± 10.1  50.7 ± 13.4  61.7 ± 19.3 | 69.2 ± 15.5  71.2 ± 21.6  83.4 ± 17.8 | 78.8 ± 14.7  91.3 ± 20.6  95.1 ± 23.4 | < 0.001  < 0.001  < 0.001 |
| A wave, cm/s  *- JHS Visit 1*  *- ARIC Visit 5*  *- ARIC Visit 7* | 75.5 ± 16.9  80.6 ± 16.3  90.3 ± 17.9 | 79.9 ± 17.2  84.6 ± 17.1  94.9 ± 19.3 | 88.4 ± 16.5  91.2 ± 19.9  100.4 ± 20.1 | 80.8 ± 14.6  84.3 ± 17.8  103.3 ± 20.8 | 75.6 ± 18.3  74.7 ± 22.3  67.6 ± 25.0 | 69.1 ± 16.1  65.6 ± 16.2  57.6 ± 12.7 | < 0.001  < 0.001  < 0.001 |
| E/A ratio  *- JHS Visit 1*  *- ARIC Visit 5*  *- ARIC Visit 7* | 1.01 ± 0.21  0.85 ± 0.17  0.84 ± 0.19 | 0.96 ± 0.28  0.81 ± 0.16  0.79 ± 0.16 | 0.69 ± 0.13  0.64 ± 0.11  0.66 ± 0.12 | 0.72 ± 0.16  0.61 ± 0.12  0.60 ± 0.15 | 0.93 ± 0.29  0.98 ± 0.25  1.38 ± 0.61 | 1.19 ± 0.24  1.43 ± 0.33  1.51 ± 0.27 | < 0.001  < 0.001  < 0.001 |
| PASP, mmHg  *- JHS Visit 1*  *- ARIC Visit 5*  *- ARIC Visit 7* | 21.9 ± 6.5  28.0 ± 5.8  32.4 ± 7.6 | 23.3 ± 7.2  27.5 ± 4.8  33.2 ± 7.1 | 21.7 ± 6.3  27.1 ± 5.8  32.5 ± 8.8 | 20.8 ± 5.5  23.8 ± 2.2  33.4 ± 12.9 | 26.4 ± 5.3  32.1 ± 6.2  47.8 ± 17.7 | 24.0 ± 6.8  33.0 ± 10.4  39.3 ± 9.8 | 0.045  < 0.001  < 0.001 |
| LA dimension, cm  *- JHS Visit 1*  *- ARIC Visit 5*  *- ARIC Visit 7* | 3.28 ± 0.62  3.34 ± 0.45  3.33 ± 0.51 | 3.24 ± 0.52  3.30 ± 0.43  3.26 ± 0.51 | 3.25 ± 0.55  3.37 ± 0.50  3.28 ± 0.60 | 3.20 ± 0.64  3.43 ± 0.49  3.48 ± 0.48 | 3.16 ± 0.76  3.42 ± 0.47  3.29 ± 0.52 | 3.39 ± 0.57  3.61 ± 0.50  3.74 ± 0.55 | 0.73  0.06  0.02 |

Abbreviations: ARIC, Atherosclerosis Risk in Communities; body surface area; eGFR, estimated glomerular filtration rate; JHS, Jackson Heart Study; LA, left atrial; LV, left ventricular; PASP, pulmonary artery systolic pressure.

**Supplemental Table 4.** Association between trajectory of cardiac function and risk of incident heart failure in 747 shared ARIC and JHS participants

| **Trajectory** | **N (%)** | **Heart failure events** | **Rate per 100 person-years (95% CI)** | **Model 1** | | **Model 2** | |
| --- | --- | --- | --- | --- | --- | --- | --- |
|  |  |  |  | **Hazard ratio**  **(95% CI)** | ***p*-value** | **Hazard ratio**  **(95% CI)** | ***p*-value** |
| Pink | 375 (50) | 21 | 0.5 (0.3-0.8) | *reference* | | *reference* | |
| Light green | 130 (17) | 7 | 0.5 (0.2-1.0) | 1.02 (0.43-2.42) | 0.97 | 1.00 (0.41-2.47) | 0.99 |
| Red | 168 (22) | 17 | 1.0 (0.6-1.6) | 1.97 (1.04-3.73) | 0.04 | 2.07 (1.08-3.99) | 0.03 |
| Dark green | 29 (4) | 5 | 1.9 (0.8-4.5) | 2.78 (1.02-7.56) | 0.045 | 2.41 (0.87-6.71) | 0.09 |
| Orange | 17 (2) | 4 | 2.3 (0.9-6.1) | 4.21 (1.42-12.4) | 0.009 | 3.61 (1.18-11.0) | 0.02 |
| Blue | 28 (4) | 6 | 2.5 (1.1-5.5) | 3.86 (1.55-9.63) | 0.004 | 4.14 (1.64-10.5) | 0.003 |

Hazard ratio for heart failure from Cox proportional hazard models. Model 1 adjusted for age and sex. Model 2 adjusted for age, sex, obesity, hypertension, diabetes, chronic kidney disease, coronary heart disease. 52 shared ARIC and JHS participants did not have follow-up data and were excluded from this analysis. Abbreviations: ARIC, Atherosclerosis Risk in Communities; CI, confidence interval; JHS, Jackson Heart Study.

**Supplemental Table 5.** Clinical and echocardiographic characteristics of 4,419 ARIC participants overall and according to predicted trajectory

|  | **Overall** | **Pink** | **Light green** | **Red** | **Dark green** | **Orange** | **Blue** | ***p*-value** |
| --- | --- | --- | --- | --- | --- | --- | --- | --- |
| *Demographics* | | | | | | | | |
| N (%) | 4,419 | 2,201 (50) | 676 (15) | 942 (21) | 162 (4) | 90 (2) | 348 (8) |  |
| Age, mean ± SD, years | 75.3 ± 5.1 | 75.2 ± 4.9 | 74.0 ± 4.7 | 76.4 ± 5.4 | 75.5 ± 4.8 | 78.9 ± 4.8 | 74.8 ± 4.7 | < 0.001 |
| Male sex, n (%) | 1,845 (42) | 940 (43) | 212 (31) | 421 (45) | 86 (53) | 51 (57) | 135 (39) | < 0.001 |
| Black, n (%) | 394 (9) | 185 (8) | 67 (10) | 92 (10) | 24 (15) | 6 (7) | 20 (6) | 0.02 |
| Field center, n (%) | | | | | | | | < 0.001 |
| Forsyth County, North Carolina | 1,217 (28) | 654 (30) | 191 (28) | 185 (20) | 36 (22) | 24 (27) | 127 (37) |  |
| Jackson, Mississippi | 291 (7) | 140 (6) | 47 (7) | 72 (8) | 18 (11) | 3 (3) | 11 (3) |  |
| Minneapolis, Minnesota | 1,505 (34) | 802 (36) | 192 (28) | 260 (28) | 56 (35) | 40 (44) | 155 (45) |  |
| Washington County, Maryland | 1,406 (32) | 605 (28) | 246 (36) | 425 (45) | 52 (32) | 23 (26) | 55 (16) |  |
| *Clinical covariates* | | | | | | | | |
| Current smoking, n (%) | 266 (6) | 135 (6) | 49 (8) | 42 (5) | 13 (8) | 4 (5) | 23 (7) | 0.18 |
| Any prior smoking, n (%) | 2,718 (62) | 1,393 (63) | 407 (60) | 567 (60) | 101 (62) | 58 (64) | 192 (55) | 0.06 |
| Obesity, n (%) | 1,372 (31) | 703 (32) | 197 (29) | 312 (34) | 55 (34) | 28 (32) | 77 (22) | 0.003 |
| Hypertension, n (%) | 3,539 (80) | 1,717 (78) | 549 (81) | 788 (84) | 144 (89) | 72 (80) | 269 (77) | < 0.001 |
| Diabetes, n (%) | 1,555 (35) | 752 (34) | 248 (37) | 359 (38) | 73 (45) | 28 (31) | 95 (27) | < 0.001 |
| Chronic kidney disease, n (%) | 1,134 (26) | 541 (25) | 156 (23) | 278 (30) | 41 (26) | 29 (33) | 89 (26) | 0.02 |
| Atrial fibrillation, n (%) | 167 (4) | 82 (4) | 24 (4) | 27 (3) | 5 (3) | 9 (10) | 20 (6) | 0.01 |
| Coronary heart disease, n (%) | 547 (13) | 271 (13) | 48 (7) | 118 (13) | 38 (24) | 23 (26) | 49 (14) | < 0.001 |
| BMI, mean ± SD, kg/m^2^ | 28.2 ± 5.3 | 28.2 ± 5.5 | 28.2 ± 5.1 | 28.7 ± 5.3 | 28.5 ± 5.1 | 27.7 ± 5.2 | 26.8 ± 5.2 | < 0.001 |
| Systolic BP, mean ± SD, mmHg | 130 ± 18 | 129 ± 17 | 130 ± 19 | 130 ± 17 | 134 ± 16 | 128 ± 20 | 130 ± 18 | 0.01 |
| Diastolic BP, mean ± SD, mmHg | 66 ± 10 | 66 ± 10 | 66 ± 10 | 68 ± 11 | 70 ± 11 | 62 ± 11 | 64 ± 10 | < 0.001 |
| Heart rate, mean ± SD, beats/min | 62 ± 10 | 61 ± 9 | 62 ± 9 | 65 ± 11 | 66 ± 10 | 58 ± 9 | 57 ± 8 | < 0.001 |

Abbreviations: ARIC, Atherosclerosis Risk in Communities; BMI, body mass index; BP, blood pressure; body surface area; CRP, C-reactive protein; eGFR, estimated glomerular filtration rate; GLS, global longitudinal strain; HDL, high density lipoprotein; Hs, high-sensitivity; LA, left atrial; LDL, low density lipoprotein; LV, left ventricular; NT-proBNP, N-terminal pro-B-type natriuretic peptide; PASP, pulmonary artery systolic pressure

**Supplemental Table 5 (continued).** Clinical and echocardiographic characteristics of 4,419 ARIC participants overall and according to predicted trajectory

|  | **Overall** | **Pink** | **Light green** | **Red** | **Dark green** | **Orange** | **Blue** | ***p*-value** |
| --- | --- | --- | --- | --- | --- | --- | --- | --- |
| *Laboratory values* | | | | | | | | |
| eGFR, mean ± SD, ml/min/1.73m^2^ | 70 ± 16 | 70 ± 16 | 72 ± 16 | 69 ± 16 | 70 ± 16 | 66 ± 16 | 70 ± 16 | < 0.001 |
| LDL cholesterol, median [IQR], mg/dL | 102 [80-125] | 102 [80-127] | 104 [80-126] | 102 [78-126] | 99 [82-120] | 90 [69-121] | 103 [84-125] | 0.15 |
| HDL cholesterol, median [IQR], mg/dL | 51 [42-60] | 51 [42-61] | 52 [43-61] | 49 [41-58] | 49 [41-57] | 48 [42-60] | 53 [44-64] | < 0.001 |
| HsCRP, median [IQR], mg/L | 1.9 [0.9-3.9] | 1.9 [0.9-4.0] | 1.9 [0.9-3.9] | 2.0 [1.0-4.1] | 2.1 [1.0-4.5] | 1.5 [0.8-3.4] | 1.6 [0.8-3.2] | 0.01 |
| NT-proBNP, median [IQR], ng/mL | 125 [68-233] | 122 [69-221] | 105 [56-189] | 119 [65-227] | 146 [71-332] | 224 [103-524] | 182 [102-339] | < 0.001 |
| Hs Troponin T, median [IQR], ng/L | 1.0 [0.7-1.5] | 1.0 [0.7-1.5] | 0.9 [0.6-1.3] | 1.1 [0.8-1.6] | 1.1 [0.8-1.7] | 1.3 [0.9-2.1] | 1.0 [0.7-1.5] | < 0.001 |
| *Echocardiographic findings, mean ± SD* | | | | | | | | |
| LV mean wall thickness, cm | 0.98 ± 0.13 | 0.97 ± 0.13 | 0.98 ± 0.13 | 1.00 ± 0.13 | 1.01 ± 0.15 | 1.01 ± 0.18 | 0.95 ± 0.13 | < 0.001 |
| LV relative wall thickness, cm | 0.42 ± 0.07 | 0.42 ± 0.07 | 0.43 ± 0.07 | 0.44 ± 0.08 | 0.42 ± 0.09 | 0.43 ± 0.09 | 0.41 ± 0.07 | < 0.001 |
| LV mass index, g/m^2^ | 79 ± 19 | 78 ± 18 | 77 ± 18 | 80 ± 18 | 86 ± 26 | 86 ± 27 | 78 ± 19 | < 0.001 |
| LV end-diastolic volume, mL | 81 ± 23 | 81 ± 23 | 75 ± 20 | 80 ± 23 | 95 ± 35 | 89 ± 26 | 81 ± 23 | < 0.001 |
| LV end-systolic volume, mL | 28 ± 11 | 28 ± 10 | 24 ± 8 | 29 ± 11 | 41 ± 23 | 32 ± 13 | 27 ± 10 | < 0.001 |
| LV ejection fraction, % | 66.0 ± 5.8 | 66.0 ± 5.2 | 68.6 ± 5.2 | 65.1 ± 5.8 | 58.9 ± 9.1 | 64.5 ± 7.1 | 67.4 ± 5.2 | < 0.001 |
| GLS, % | -18.2 ± 2.4 | -18.4 ± 2.2 | -18.6 ± 2.3 | -17.7 ± 2.5 | -16.5 ± 2.8 | -18.2 ± 2.4 | -18.7 ± 2.4 | < 0.001 |
| E wave, cm/s | 65.7 ± 17.0 | 68.0 ± 15.2 | 67.8 ± 15.6 | 54.4 ± 12.5 | 53.2 ± 12.9 | 76.9 ± 21.3 | 81.0 ± 19.5 | < 0.001 |
| A wave, cm/s | 80.6 ± 19.1 | 79.3 ± 17.9 | 83.5 ± 17.8 | 88.1 ± 18.6 | 86.1 ± 19.2 | 74.6 ± 20.2 | 61.9 ± 14.3 | < 0.001 |
| E/A ratio | 0.84 ± 0.23 | 0.87 ± 0.15 | 0.82 ± 0.17 | 0.62 ± 0.08 | 0.62 ± 0.10 | 1.06 ± 0.24 | 1.33 ± 0.23 | < 0.001 |
| E/e’ septal | 12.2 ± 4.2 | 12.3 ± 4.0 | 12.3 ± 3.8 | 11.2 ± 3.7 | 11.6 ± 4.4 | 15.0 ± 7.3 | 13.5 ± 5.6 | < 0.001 |
| E/e’ lateral | 10.2 ± 3.7 | 10.3 ± 3.6 | 10.5 ± 3.7 | 9.4 ± 3.3 | 9.6 ± 3.4 | 11.8 ± 5.5 | 11.0 ± 4.5 | < 0.001 |
| PASP, mmHg | 27.8 ± 5.3 | 27.7 ± 5.3 | 28.0 ± 5.3 | 27.0 ± 4.9 | 26.0 ± 4.6 | 29.2 ± 6.2 | 29.5 ± 6.3 | < 0.001 |
| LA dimension, cm | 3.5 ± 0.5 | 3.5 ± 0.5 | 3.4 ± 0.5 | 3.5 ± 0.5 | 3.6 ± 0.5 | 3.7 ± 0.5 | 3.6 ± 0.5 | < 0.001 |
| LA volume index, mL/m^2^ | 24.9 ± 7.4 | 25.0 ± 7.2 | 24.3 ± 7.1 | 23.4 ± 6.8 | 24.8 ± 7.4 | 30.2 ± 8.4 | 27.9 ± 9.4 | < 0.001 |

Abbreviations: ARIC, Atherosclerosis Risk in Communities; BMI, body mass index; BP, blood pressure; body surface area; CRP, C-reactive protein; eGFR, estimated glomerular filtration rate; GLS, global longitudinal strain; HDL, high density lipoprotein; Hs, high-sensitivity; LA, left atrial; LDL, low density lipoprotein; LV, left ventricular; NT-proBNP, N-terminal pro-B-type natriuretic peptide; PASP, pulmonary artery systolic pressure

**Supplemental Table 6.** Mean change in echocardiographic parameters between ARIC Visit 5 and 7 according to trajectory of cardiac function

| **Variable** |  | **Pink** | **Light green** | **Red** | **Dark green** | **Orange** | **Blue** |
| --- | --- | --- | --- | --- | --- | --- | --- |
| LV mean wall thickness, cm | N | 1,164 | 350 | 364 | 56 | 49 | 177 |
|  | Visit 5 | 1.0 ± 0.1 | 1.0 ± 0.1 | 1.0 ± 0.1 | 1.0 ± 0.2 | 1.0 ± 0.1 | 0.9 ± 0.1 |
|  | Visit 7 | 1.0 ± 0.2 | 1.0 ± 0.1 | 1.0 ± 0.1 | 1.1 ± 0.2 | 1.0 ± 0.1 | 1.0 ± 0.2 |
|  | Delta | 0.05 ± 0.1 | 0.05 ± 0.1 | 0.06 ± 0.1 | 0.08 ± 0.1 | 0.02 ± 0.1 | 0.03 ± 0.1 |
|  | *p* vs. Pink | *NA* | 0.47 | 0.12 | 0.04 | 0.07 | 0.17 |
| LV relative wall thickness, cm | N | 1,157 | 345 | 359 | 54 | 49 | 176 |
|  | Visit 5 | 0.42 ± 0.06 | 0.43 ± 0.07 | 0.43 ± 0.07 | 0.42 ± 0.09 | 0.42 ± 0.07 | 0.41 ± 0.06 |
|  | Visit 7 | 0.45 ± 0.08 | 0.47 ± 0.08 | 0.46 ± 0.09 | 0.43 ± 0.09 | 0.42 ± 0.09 | 0.43 ± 0.09 |
|  | Delta | 0.03 ± 0.08 | 0.04 ± 0.08 | 0.03 ± 0.09 | 0.02 ± 0.10 | - 0.00 ± 0.09 | 0.02 ± 0.10 |
|  | *p* vs. Pink | *NA* | 0.08 | 0.93 | 0.35 | 0.01 | 0.28 |
| LV mass index, g/m^2^ | N | 1,145 | 344 | 357 | 52 | 48 | 176 |
|  | Visit 5 | 76 ± 17 | 75 ± 16 | 78 ± 16 | 87 ± 30 | 84 ± 18 | 76 ± 17 |
|  | Visit 7 | 80 ± 19 | 77 ± 18 | 83 ± 18 | 101 ± 34 | 89 ± 22 | 79 ± 19 |
|  | Delta | 4.0 ± 14 | 2.2 ± 14 | 5.6 ± 15 | 14 ± 23 | 5.3 ± 16 | 3.2 ± 14 |
|  | *p* vs. Pink | *NA* | 0.048 | 0.06 | <0.001 | 0.54 | 0.54 |
| LV end-diastolic volume, mL | N | 1,127 | 334 | 344 | 54 | 49 | 177 |
|  | Visit 5 | 82 ± 22 | 74 ± 20 | 83 ± 23 | 102 ± 35 | 91 ± 29 | 83 ± 23 |
|  | Visit 7 | 85 ± 23 | 76 ± 21 | 87 ± 25 | 112 ± 41 | 101 ± 29 | 84 ± 24 |
|  | Delta | 2.9 ± 14 | 2.7 ± 12 | 4.6 ± 16 | 11 ± 26 | 10 ± 15 | 1.1 ± 12 |
|  | *p* vs. Pink | *NA* | 0.78 | 0.05 | <0.001 | 0.001 | 0.12 |
| LV end-systolic volume, mL | N | 1,127 | 334 | 344 | 54 | 49 | 177 |
|  | Visit 5 | 28 ± 10 | 23 ± 8 | 29 ± 11 | 45 ± 25 | 32 ± 13 | 28 ± 10 |
|  | Visit 7 | 31 ± 11 | 26 ± 9 | 34 ± 16 | 57 ± 35 | 43 ± 20 | 30 ± 12 |
|  | Delta | 3.1 ± 8 | 2.3 ± 6 | 4.7 ± 12 | 13 ± 26 | 11 ± 14 | 2.8 ± 8 |
|  | *p* vs. Pink | *NA* | 0.19 | 0.004 | <0.001 | <0.001 | 0.70 |
| LV ejection fraction, % | N | 1,174 | 352 | 369 | 59 | 50 | 180 |
|  | Visit 5 | 65.9 ± 4.9 | 68.3 ± 4.9 | 64.9 ± 62.2 | 58.4 ± 9.0 | 65.2 ± 5.6 | 67.0 ± 5.0 |
|  | Visit 7 | 63.9 ± 5.6 | 67.1 ± 5.2 | 62.3 ± 7.1 | 52.6 ±11.7 | 58.3 ± 10.0 | 64.2 ± 5.9 |
|  | Delta | -2.0 ± 5.8 | -1.2 ± 6.4 | -2.6 ± 6.9 | -5.8 ± 10.2 | -6.9 ± 8.4 | -2.8 ± 6.1 |
|  | *p* vs. Pink | *NA* | 0.03 | 0.12 | <0.001 | <0.001 | 0.14 |

Abbreviations: ARIC, Atherosclerosis Risk in Communities; GLS, global longitudinal strain; LA, left atrial; LV, left ventricular; PASP, pulmonary artery systolic pressure.

**Supplemental Table 6 (continued).** Mean change in echocardiographic parameters between ARIC Visit 5 and 7 according to trajectory of cardiac function

| **Variable** |  | **Pink** | **Light green** | **Red** | **Dark green** | **Orange** | **Blue** |
| --- | --- | --- | --- | --- | --- | --- | --- |
| GLS, % | N | 1,115 | 334 | 338 | 52 | 47 | 177 |
|  | Visit 5 | -18.4 ± 2.2 | -18.7 ± 2.2 | -17.7 ± 2.3 | -16.9 ± 2.7 | -18.2 ± 2.3 | -18.6 ± 2.3 |
|  | Visit 7 | -17.9 ± 2.4 | -18.3 ± 2.2 | -17.0 ± 2.5 | -15.3 ± 3.2 | -16.7 ± 3.2 | -18.3 ± 2.8 |
|  | Delta | -0.5 ± 2.7 | -0.3 ± 2.7 | -0.6 ± 3.0 | -1.6 ± 2.9 | -1.5 ± 3.5 | -0.4 ± 2.8 |
|  | *p* vs. Pink | *NA* | 0.21 | 0.57 | 0.01 | 0.02 | 0.45 |
| PASP, mmHg | N | 562 | 183 | 156 | 19 | 23 | 103 |
|  | Visit 5 | 27.1 ± 4.5 | 27.4 ± 4.4 | 26.7 ± 4.3 | 25.6 ± 5.3 | 27.2 ± 5.2 | 28.3 ± 5.1 |
|  | Visit 7 | 31.9 ± 7.8 | 32.4 ± 7.1 | 30.2 ± 6.8 | 31.9 ± 7.1 | 35.3 ± 12.6 | 35.3 ± 7.3 |
|  | Delta | 4.8 ± 7.5 | 5.0 ± 6.8 | 3.6 ± 6.6 | 6.3 ± 6.2 | 8.1 ± 10.8 | 8.1 ± 10.8 |
|  | *p* vs. Pink | *NA* | 0.66 | 0.08 | 0.38 | 0.03 | 0.01 |
| E wave, cm/s | N | 1,165 | 351 | 364 | 57 | 50 | 180 |
|  | Visit 5 | 67 ± 15 | 68 ± 15 | 54 ± 12 | 52 ± 12 | 71 ± 20 | 78 ± 18 |
|  | Visit 7 | 76 ± 20 | 75 ± 19 | 62 ± 17 | 64 ± 21 | 88 ± 22 | 90 ± 25 |
|  | Delta | 9 ± 18 | 8 ± 19 | 8 ± 16 | 12 ± 20 | 18 ± 18 | 13 ± 21 |
|  | *p* vs. Pink | *NA* | 0.31 | 0.67 | 0.18 | 0.001 | 0.01 |
| A wave, cm/s | N | 1,123 | 344 | 356 | 53 | 44 | 157 |
|  | Visit 5 | 78 ± 18 | 82 ± 16 | 85 ± 16 | 82 ± 16 | 76 ± 20 | 60 ± 13 |
|  | Visit 7 | 88 ± 20 | 94 ± 19 | 97 ± 20 | 91 ± 19 | 72 ± 23 | 66 ± 18 |
|  | Delta | 10 ± 15 | 12 ± 16 | 12 ± 14 | 8 ± 17 | - 5 ± 20 | 6 ± 17 |
|  | *p* vs. Pink | *NA* | 0.03 | 0.045 | 0.26 | <0.001 | <0.001 |
| E/A ratio | N | 1,116 | 344 | 352 | 52 | 44 | 157 |
|  | Visit 5 | 0.88 ± 0.17 | 0.84 ± 0.18 | 0.64 ± 0.10 | 0.63 ± 0.12 | 0.94 ± 0.26 | 1.30 ± 0.28 |
|  | Visit 7 | 0.86 ± 0.17 | 0.80 ± 0.16 | 0.64 ± 0.13 | 0.68 ± 0.17 | 1.30 ± 0.47 | 1.38 ± 0.33 |
|  | Delta | - 0.02 ± 0.23 | - 0.04 ± 0.21 | - 0.00 ± 0.18 | 0.05 ± 0.19 | 0.36 ± 0.58 | 0.08 ± 0.49 |
|  | *p* vs. Pink | *NA* | 0.27 | 0.28 | 0.07 | <0.001 | <0.001 |
| LA volume index, mL/m^2^ | N | 1133 | 345 | 355 | 55 | 49 | 178 |
|  | Visit 5 | 24.6 ± 6.7 | 23.5 ± 6.4 | 22.5 ± 6.3 | 24.3 ± 6.9 | 28.4 ± 8.3 | 26.9 ± 6.8 |
|  | Visit 7 | 27.0 ± 7.8 | 25.5 ± 7.5 | 25.7 ± 7.5 | 27.4 ± 8.7 | 34.4 ± 13.1 | 31.6 ± 9.7 |
|  | Delta | 2.4 ± 6.5 | 2.0 ± 5.9 | 3.1 ± 6.6 | 3.1 ± 6.0 | 6.1 ± 9.2 | 4.7 ± 7.1 |
|  | *p* vs. Pink | *NA* | 0.35 | 0.07 | 0.45 | <0.001 | <0.001 |

Abbreviations: ARIC, Atherosclerosis Risk in Communities; GLS, global longitudinal strain; LA, left atrial; LV, left ventricular; PASP, pulmonary artery systolic pressure.

**Supplemental Table 7.** Association between trajectory and risk of incident heart failure in 4,439 ARIC-participants and according to trajectory derived from participants with 3 echocardiograms

| **Trajectory** | **Prevalence**  **N (%)** | **Any heart Failure** | | | **HFpEF** | | | **HFrEF** | | |
| --- | --- | --- | --- | --- | --- | --- | --- | --- | --- | --- |
|  |  | **Events** | **Hazard ratio**  **(95% CI)** | ***p*-value** | **Events** | **Hazard ratio**  **(95% CI)** | ***p*-value** | **Events** | **Hazard ratio**  **(95% CI)** | ***p*-value** |
| Pink | 1,853 (42) | 146 | *reference* | | 76 | *reference* | | 46 | *reference* | |
| Light green | 996 (22) | 43 | 0.63 (0.44-0.90) | 0.01 | 20 | 0.53 (0.31-0.89) | 0.02 | 13 | 0.59 (0.31-1.12) | 0.11 |
| Red | 971 (22) | 89 | 1.19 (0.91-1.55) | 0.21 | 44 | 1.17 (0.80-1.70) | 0.42 | 33 | 1.31 (0.83-2.08) | 0.24 |
| Dark green | 78 (2) | 18 | 3.55 (2.16-5.82) | <0.001 | 3 | 1.23 (0.39-3.91) | 0.73 | 13 | 7.66 (4.09-14.3) | <0.001 |
| Orange | 122 (3) | 25 | 2.11 (1.36-3.26) | 0.001 | 15 | 2.49 (1.40-4.45) | 0.002 | 10 | 2.71 (1.36-5.41) | 0.005 |
| Blue | 419 (9) | 51 | 1.72 (1.24-2.40) | 0.001 | 32 | 2.05 (1.34-3.14) | 0.001 | 11 | 1.25 (0.64- 2.43) | 0.52 |

Hazard ratios from Cox proportional hazard models were adjusted for age, sex, obesity, hypertension, diabetes, chronic kidney disease, coronary heart disease and atrial fibrillation. 28 ARIC participants did not have follow-up data and were excluded from this analysis. Participants with a HF events with LVEF<50% or unknown LVEF at the time of HF hospitalization were censored in analyses with incident HFpEF as endpoint. Conversely, HF events with LVEF≥50% or unknown LVEF at the time of HF hospitalization were censored in analyses with incident HFrEF as endpoint.

Abbreviations: ARIC, Atherosclerosis Risk in Communities; CI, confidence interval; HF, heart failure; HFpEF, heart failure with preserved left ventricular ejection fraction; HFrEF, heart failure reduced left ventricular ejection fraction; HR, hazard ratio; JHS, Jackson Heart Study; LVEF, left ventricular ejection fraction

**Supplemental Table 8.** Incremental predictive value of predicted trajectory membership among ARIC-only participants

| **Endpoint** | **Demographics + comorbidities + LVEF + E/A-ratio** | **Demographics + comorbidities + LVEF + E/A-ratio + predicted trajectory membership** | **Difference in C statistic**  **(95% CI)** | ***p*-value** |
| --- | --- | --- | --- | --- |
|  | **C statistic (95% CI)** | **C statistic (95% CI)** |  |  |
| HF overall | 0.723 (0.696, 0.748) | 0.733 (0.708, 0.758) | 0.011 (0.003, 0.020) | 0.010 |
| HFpEF | 0.686 (0.648, 0.723) | 0.705 (0.669, 0.741) | 0.020 (0.004, 0.035) | 0.015 |
| HFrEF | 0.804 (0.766, 0.843) | 0.809 (0.771, 0.847) | 0.005 (-0.006, 0.016) | 0.414 |

Demographics = age and sex. Comorbidities = obesity (BMI > 30 kg/m^2^), hypertension, diabetes, chronic kidney disease, coronary heart disease, atrial fibrillation.

Abbreviations: BMI, body mass index; CI, confidence interval; HF, heart failure; HFpEF, heart failure with preserved left ventricular ejection fraction; HFrEF, heart failure reduced left ventricular ejection fraction; LVEF, left ventricular ejection fraction

**Supplemental Table 9.** Incremental predictive value of predicted trajectory membership among ARIC-only participants with NT-proBNP

| **Endpoint** | **Demographics + comorbidities + LVEF + E/A-ratio + NTproBNP** | **Demographics + comorbidities + LVEF + E/A-ratio + NTproBNP + predicted trajectory membership** | **Difference in C statistic**  **(95% CI)** | ***p*-value** |
| --- | --- | --- | --- | --- |
|  | **C statistic (95% CI)** | **C statistic (95% CI)** |  |  |
| HF overall | 0.761 (0.737-0.786) | 0.767 (0.743-0.791) | 0.006 (-0.002-0.013) | 0.136 |
| HFpEF | 0.732 (0.695-0.768) | 0.744 (0.708-0.779) | 0.012 (-0.001-0.025) | 0.078 |
| HFrEF | 0.824 (0.787-0.861) | 0.826 (0.789-0.864) | 0.003 (-0.007-0.013) | 0.597 |

Demographics = age and sex. Comorbidities = obesity (BMI > 30 kg/m^2^), hypertension, diabetes, chronic kidney disease, coronary heart disease, atrial fibrillation.

Abbreviations: BMI, body mass index; CI, confidence interval; HF, heart failure; HFpEF, heart failure with preserved left ventricular ejection fraction; HFrEF, heart failure reduced left ventricular ejection fraction; LVEF, left ventricular ejection fraction; NT-proBNP, N-terminal pro-B-type natriuretic peptide

**Supplemental Table 10.** Drugs and Clinical Candidates from the ChEMBL database^11^

| **Name** | **Protein** | **ChEMBL ID** | **Parent Molecule ChEMBL ID** | **Parent**  **Molecule Name** | **Parent Molecule Type** | **Max Phase** | **First Approval** | **Drug Mechanisms** |
| --- | --- | --- | --- | --- | --- | --- | --- | --- |
| Insulin-like growth factor 1 receptor | IGF1R | CHEMBL1957 | CHEMBL1743019 | FIGITUMUMAB | Antibody | 3 | None | IGF1R receptor antagonist |
|  |  |  | CHEMBL1743024 | GANITUMAB | Antibody | 3 | None | IGF1R receptor antagonist |
|  |  |  | CHEMBL1201717 | MECASERMIN RINFABATE | Protein | 4 | 2005 | IGF1R receptor agonist |
|  |  |  | CHEMBL1201716 | MECASERMIN | Protein | 4 | 2005 | IGF1R receptor agonist |
|  |  |  | CHEMBL2109356 | BIIB-022 | Antibody | 1 | None | IGF1R receptor inhibitor |
|  |  |  | CHEMBL1091644 | LINSITINIB | Small molecule | 3 | None | IGF1R receptor inhibitor |
|  |  |  | CHEMBL3989990 | ISTIRATUMAB | Antibody | 2 | None | IGF1R receptor inhibitor |
|  |  |  | CHEMBL1743006 | DALOTUZUMAB | Antibody | 2 | None | IGF1R receptor antagonist |
|  |  |  | CHEMBL2109357 | AVE-1642 | Antibody | 2 | None | IGF1R receptor antagonist |
|  |  |  | CHEMBL3545085 | XL-228 | Small molecule | 1 | None | IGF1R receptor inhibitor |
|  |  |  | CHEMBL3899477 | CONTELTINIB | Small molecule | 1 | None | IGF1R receptor inhibitor |
|  |  |  | CHEMBL551064 | AEW-541 | Small molecule | 1 | None | IGF1R receptor inhibitor |
|  |  |  | CHEMBL283120 | AXL-1717 | Small molecule | 2 | None | IGF1R receptor inhibitor |
|  |  |  | CHEMBL575448 | BMS-754807 | Small molecule | 2 | None | IGF1R receptor inhibitor |
|  |  |  | CHEMBL3545004 | PL-225B | Small molecule | 1 | None | IGF1R receptor inhibitor |
|  |  |  | CHEMBL3545025 | INSM-18 | Small molecule | 2 | None | IGF1R receptor inhibitor |
|  |  |  | CHEMBL3545156 | KW-2450 | Small molecule | 2 | None | IGF1R receptor inhibitor |
|  |  |  | CHEMBL1743064 | ROBATUMUMAB | Antibody | 2 | None | IGF1R receptor antagonist |
|  |  |  | CHEMBL1743079 | TEPROTUMUMAB | Antibody | 4 | 2020 | IGF1R receptor antagonist |
|  |  |  | CHEMBL1743001 | CIXUTUMUMAB | Antibody | 2 | None | IGF1R receptor antagonist |
| Endoglin | ENG | CHEMBL3712885 | CHEMBL2109321 | CAROTUXIMAB | Antibody | 3 | No Data |  |
| OX-2 membrane glycoprotein | CD200 | CHEMBL3712870 | CHEMBL1743067 | SAMALIZUMAB | Antibody | 2 | No Data |  |

**Supplemental Table 11.** Trajectory associated proteins annotated as potentially druggable in The Druggable Genome^12^

| **Name** | **Protein** | **Ensembl gene ID** | **Tier** | **GWAS regions** | **Small molecule** | **Biotherapeutic** | **ADME** |
| --- | --- | --- | --- | --- | --- | --- | --- |
| Fibroblast growth factor-binding protein 1 | FGFBP1 | ENSG00000137440 | Tier 3B | 0 | No | Yes | No |
| Serum albumin | ALB | ENSG00000163631 | Tier 2 | 1 | Yes | Yes | No |
| Insulin-like growth factor 1 receptor | IGF1R | ENSG00000140443 | Tier 1 | 4 | Yes | Yes | No |
| Endoglin | ENG | ENSG00000106991 | Tier 1 | 0 | No | Yes | No |
| OX-2 membrane glycoprotein | CD200 | ENSG00000091972 | Tier 1 | 0 | No | Yes | No |
| Microfibril-associated glycoprotein 4 | MFAP4 | ENSG00000166482 | Tier 3B | 0 | No | Yes | No |
| Vasorin | VASN | ENSG00000274334 |  |  |  |  |  |
|  |  | ENSG00000168140 | Tier 3B | 0 | No | Yes | No |
| Leukemia inhibitory factor receptor | LIFR | ENSG00000113594 | Tier 3A | 0 | No | Yes | No |

| **Ensembl gene ID** | The Ensembl gene identifier (from Ensembl version 75) |
| --- | --- |
| **Tier** | The priority tier for the druggable gene:   - *Tier 1:* efficacy targets of approved (1427 genes) small molecules and biotherapeutic drugs as well as clinical-phase drug candidates. - *Tier 2:* composed of 682 genes encoding targets with known bioactive drug-like small-molecule binding partners as well as those with ≥50% identity (over ≥75% of the sequence) with approved drug targets. - *Tier 3*: 2370 genes encoding secreted or extracellular proteins, proteins with more distant similarity to approved drug targets, and members of key druggable gene families not already included in tier 1 or 2 |
| **GWAS regions** | The number of GWAS LD intervals that the gene has overlapped |
| **Small molecule** | Is the protein product of the gene targeted, or predicted to be targeted, by a small molecule? |
| **Biotherapeutic** | Is the protein product of the gene targeted, or predicted to be targeted, by a biotherapeutic (Monoclonal antibody/enzyme or other protein)? |
| **ADME** | Is the protein product of the gene involved in absorption, distribution, metabolism, and excretion (ADME) of a compound? |

Abbreviations: GWAS, genome-wide association studies; LD, linkage disequilibrium

**Supplemental Figure 1.** Trajectories of cardiac function (LVEF and E/A-ratio) in 369 shared ARIC-JHS participants with 3 echocardiograms


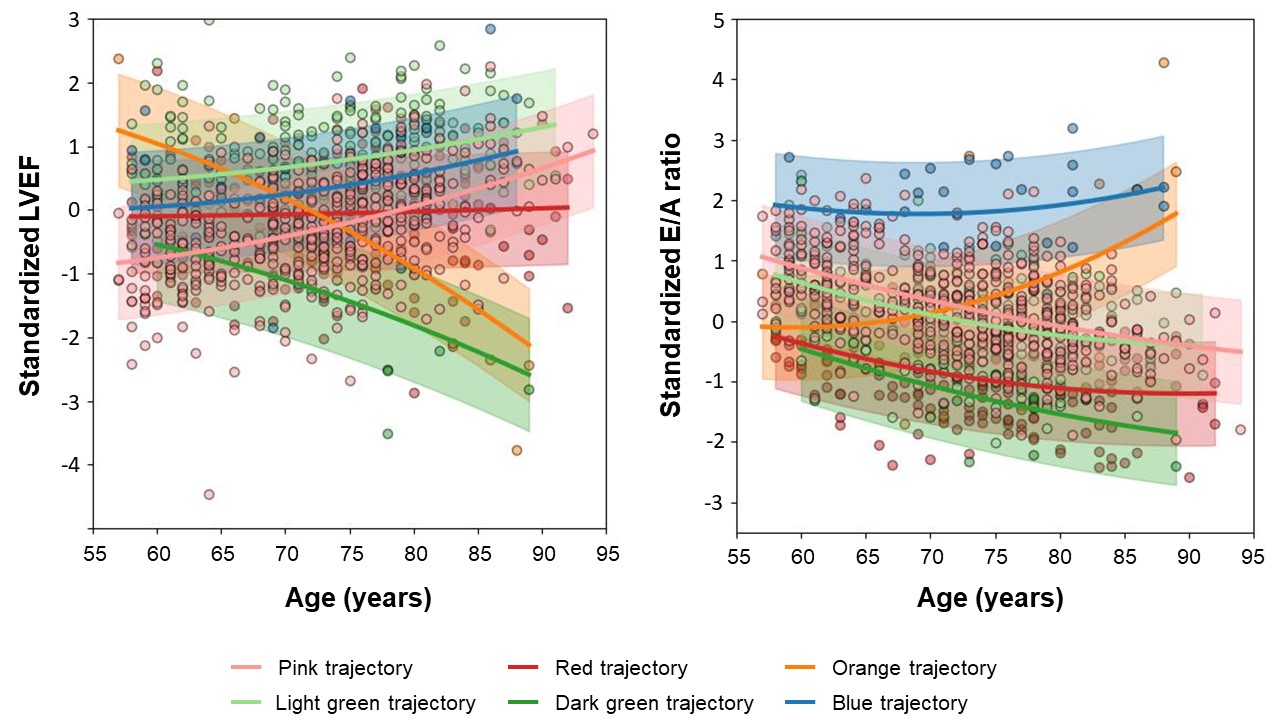


Among 369 shared ARIC-JHS participants who underwent 3 echocardiograms at JHS Visit 1 (2000-04), ARIC Visit 5 (2011-13), and ARIC Visit 7 (2018-19), we identified six trajectories of cardiac function based on LVEF, E/A ratio and age using a Bayesian nonparametric trajectory mixture modeling approach. Abbreviations: ARIC, Atherosclerosis Risk in Communities; JHS, Jackson Heart Study; LVEF, left ventricular ejection fraction

**Supplemental Figure 2.** Manhattan plot of MR associations between trajectory associated proteins and LV volumes and LVEF


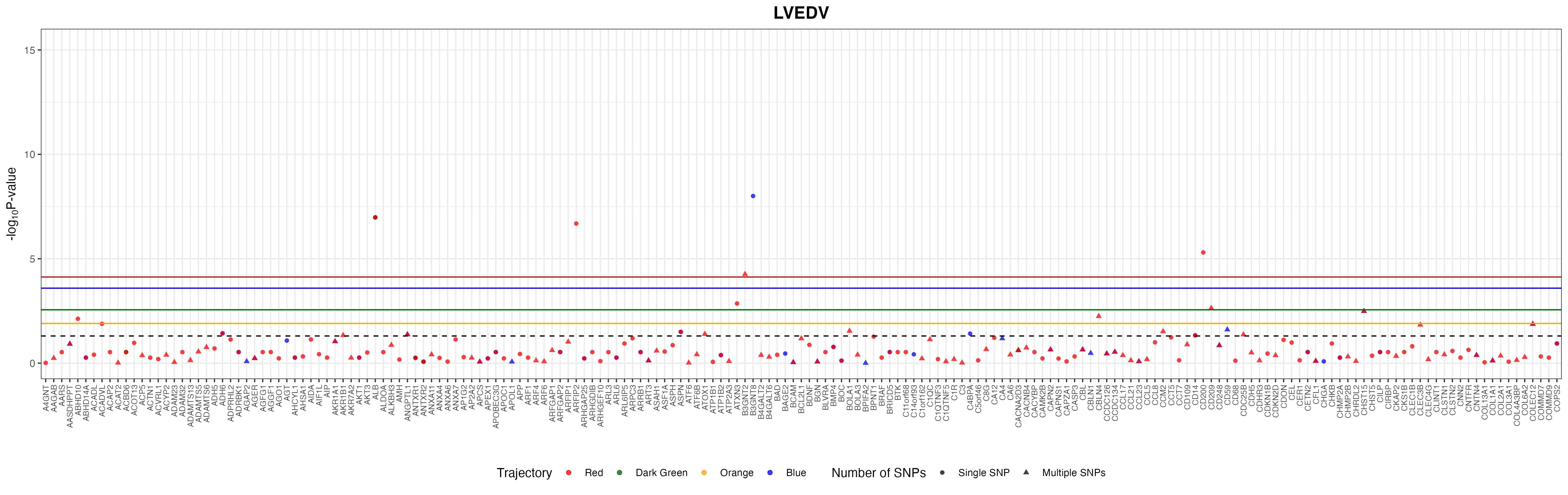


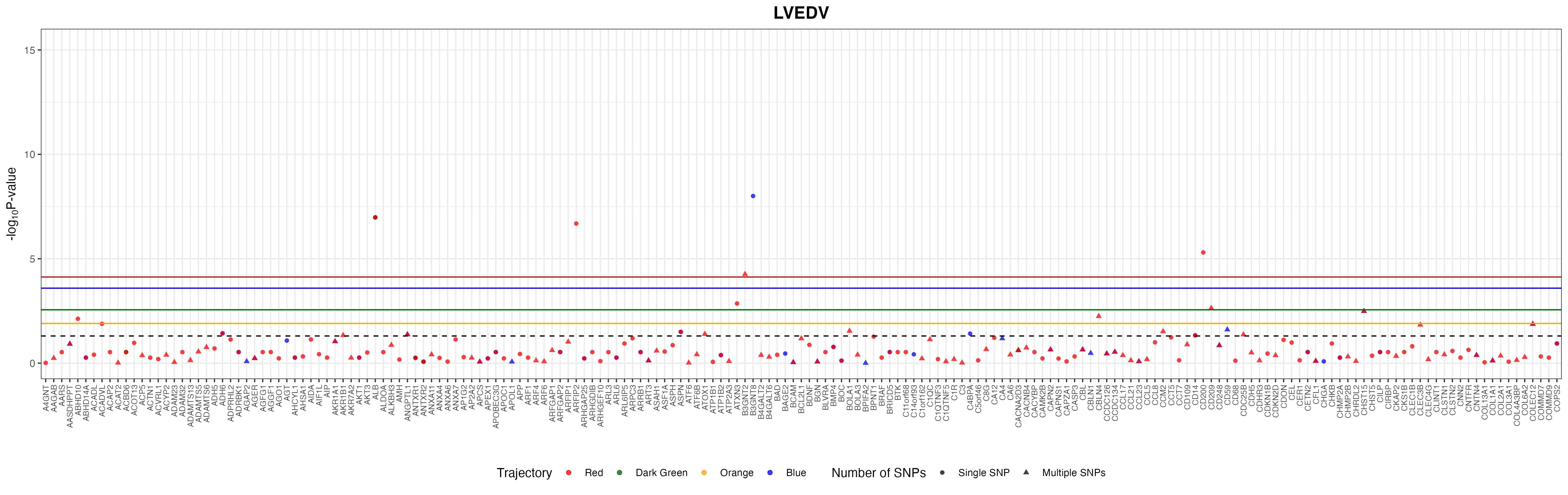


Abbreviations: LV, left ventricular; LVEDV, left ventricular end-diastolic volume; LVEF, left ventricular ejection fraction; LVESV, left ventricular end-systolic volume; MR, Mendelian Randomization; SNP, single-nucleotide polymorphism.

**Supplemental Figure 2 (continued).** Manhattan plot of MR associations between trajectory associated proteins and LV volumes and LVEF


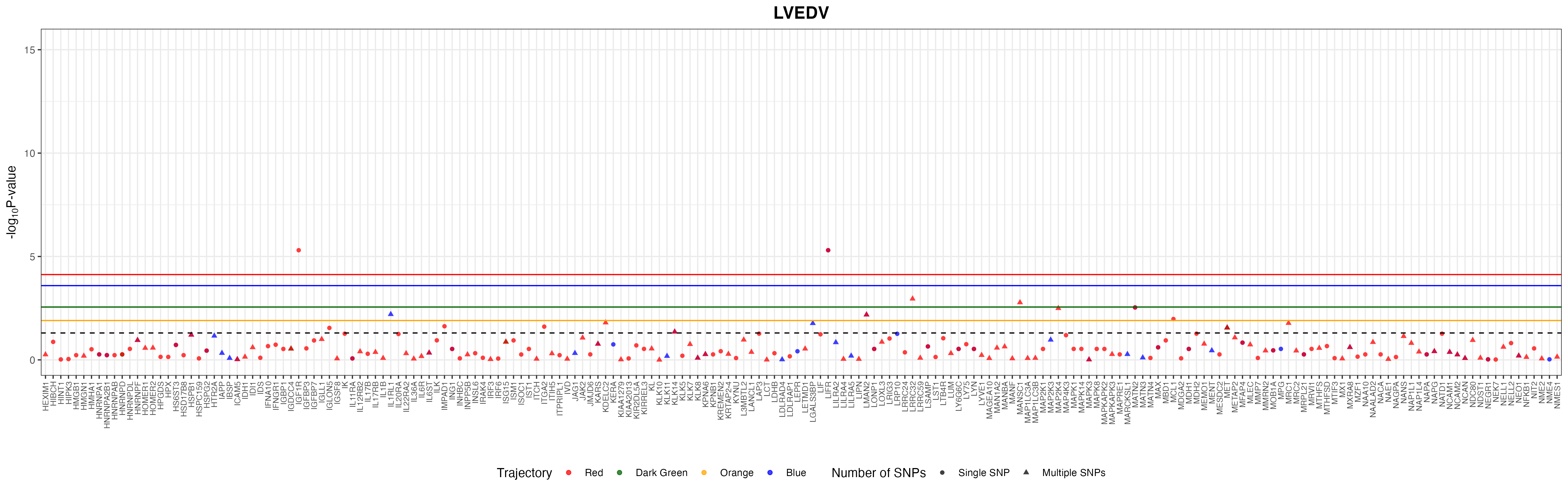


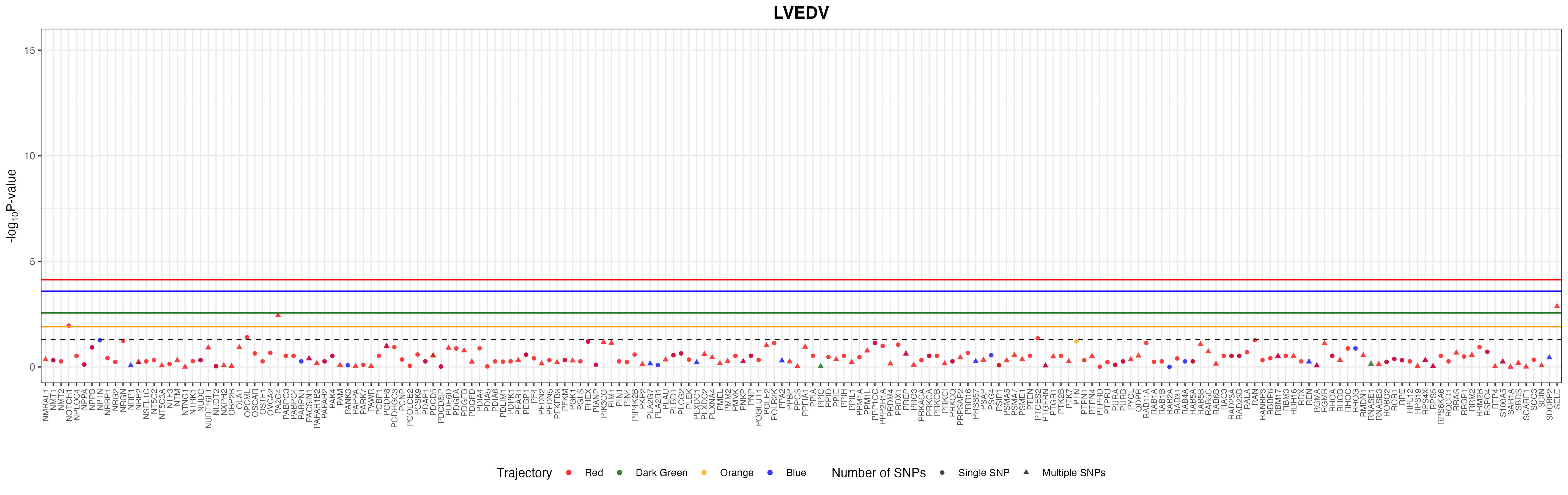


Abbreviations: LV, left ventricular; LVEDV, left ventricular end-diastolic volume; LVEF, left ventricular ejection fraction; LVESV, left ventricular end-systolic volume; MR, Mendelian Randomization; SNP, single-nucleotide polymorphism

**Supplemental Figure 2 (continued).** Manhattan plot of MR associations between trajectory associated proteins and LV volumes and LVEF


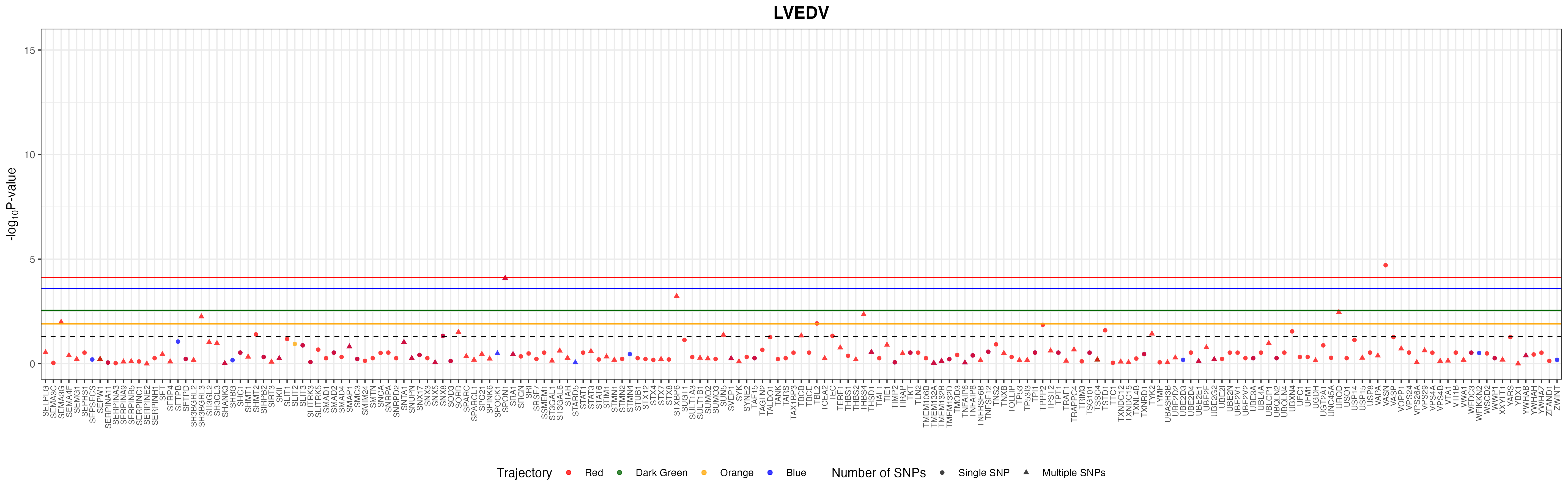


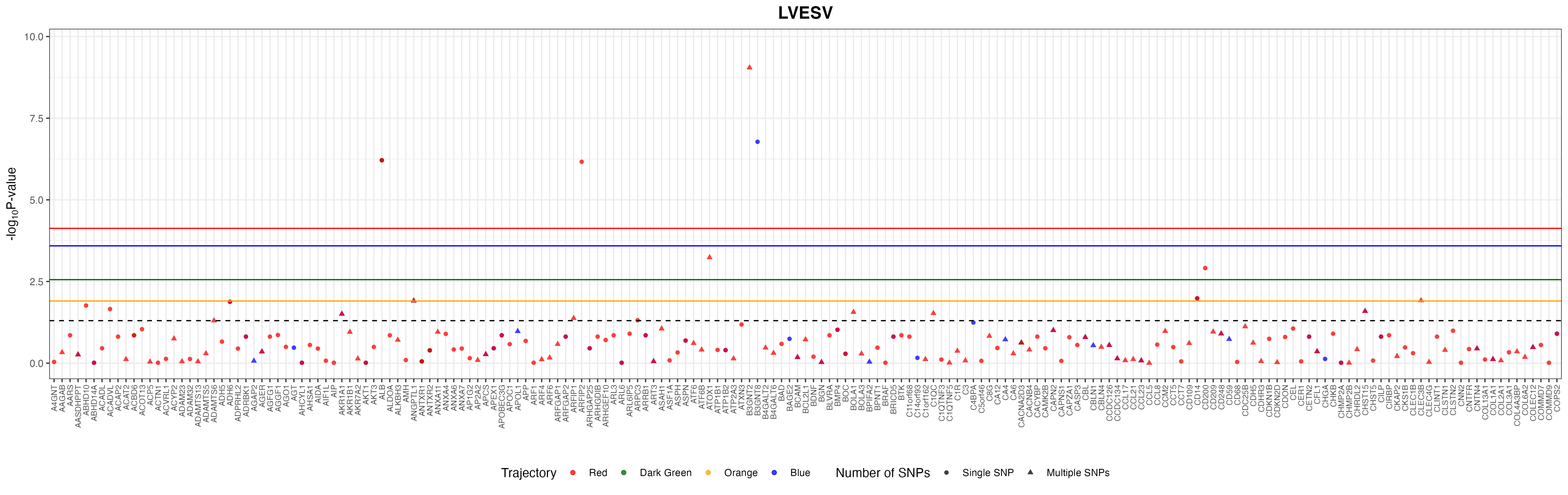


Abbreviations: LV, left ventricular; LVEDV, left ventricular end-diastolic volume; LVEF, left ventricular ejection fraction; LVESV, left ventricular end-systolic volume; MR, Mendelian Randomization; SNP, single-nucleotide polymorphism**Supplemental Figure 2 (continued).** Manhattan plot of MR associations between trajectory associated proteins and LV volumes and LVEF


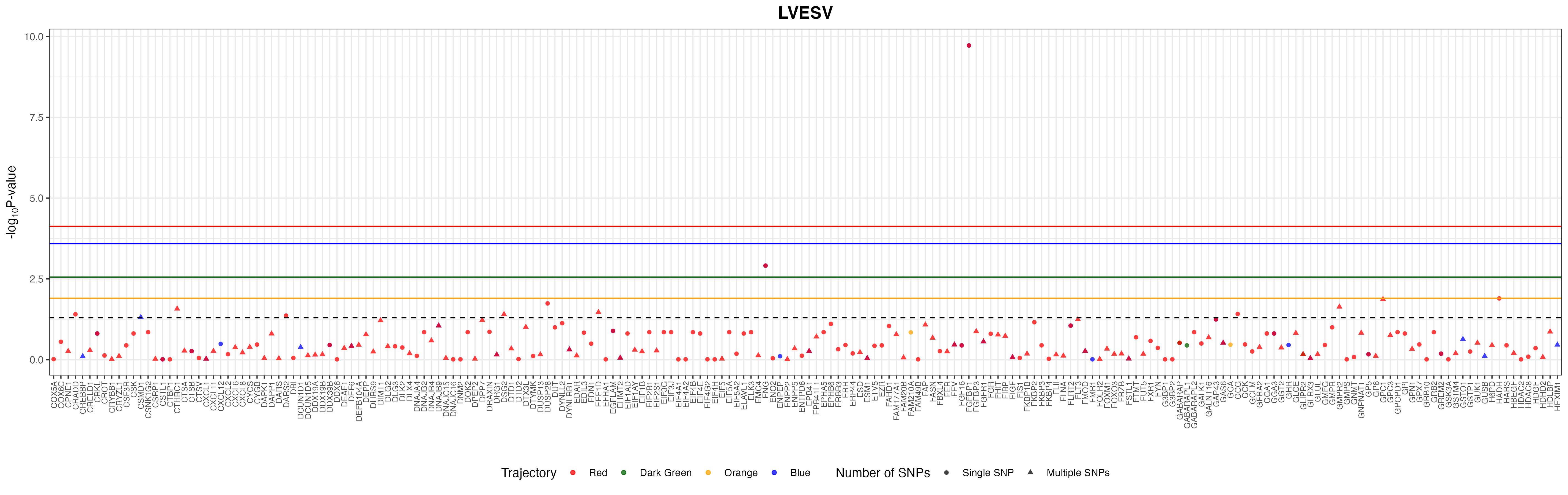


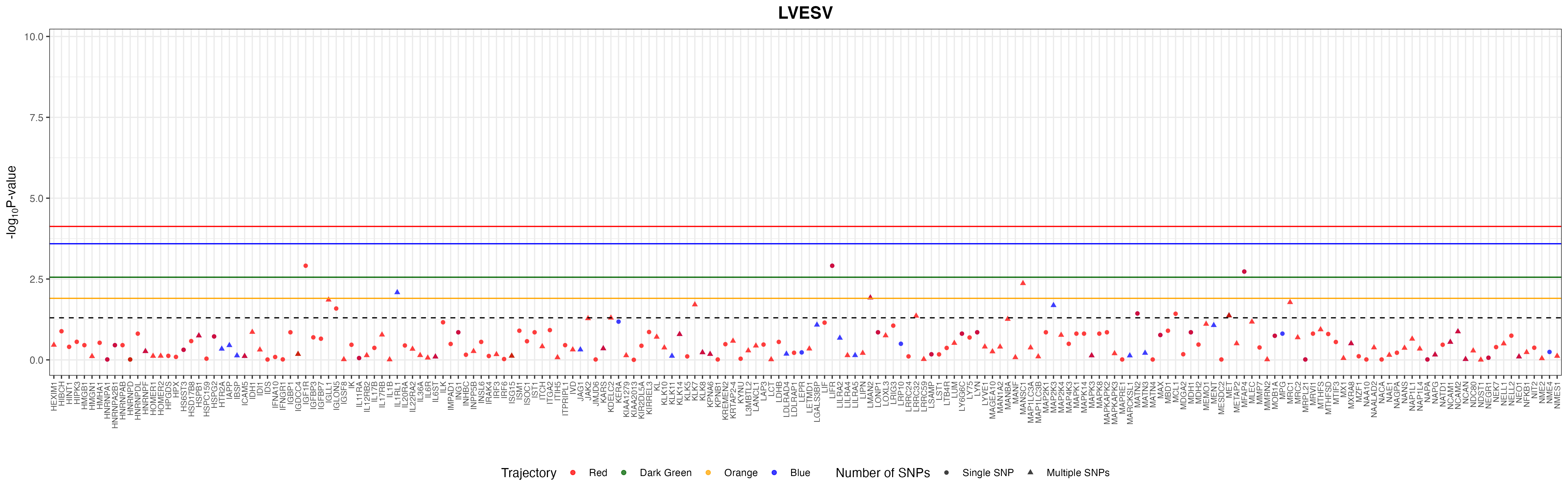


Abbreviations: LV, left ventricular; LVEDV, left ventricular end-diastolic volume; LVEF, left ventricular ejection fraction; LVESV, left ventricular end-systolic volume; MR, Mendelian Randomization; SNP, single-nucleotide polymorphism

**Supplemental Figure 2 (continued).** Manhattan plot of MR associations between trajectory associated proteins and LV volumes and LVEF


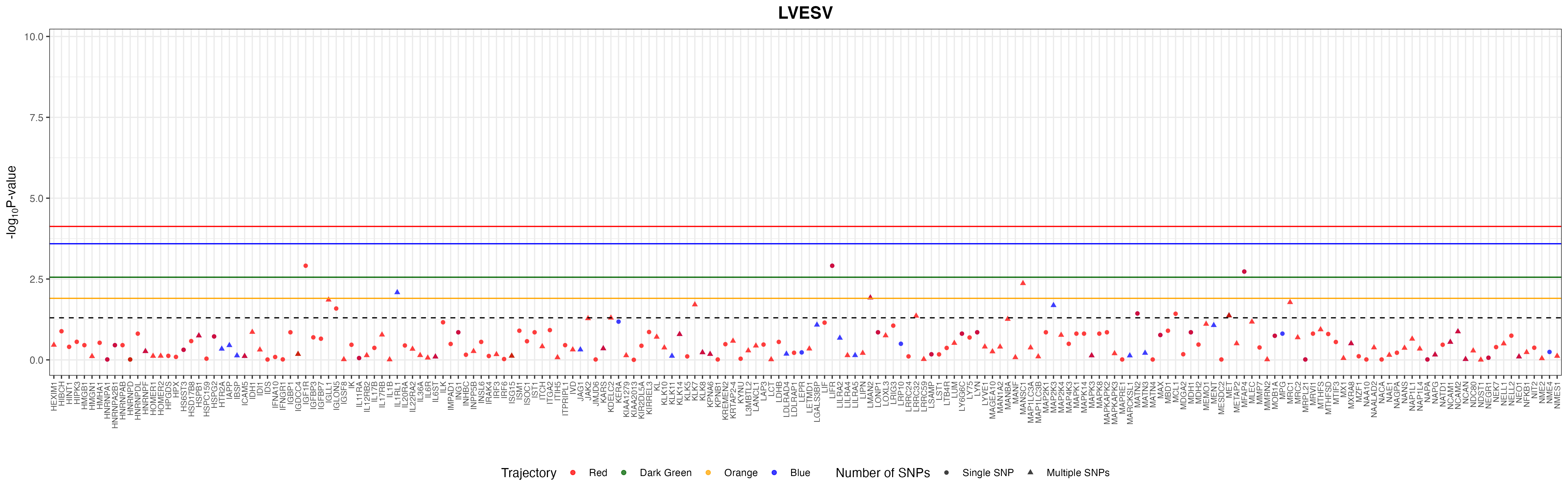


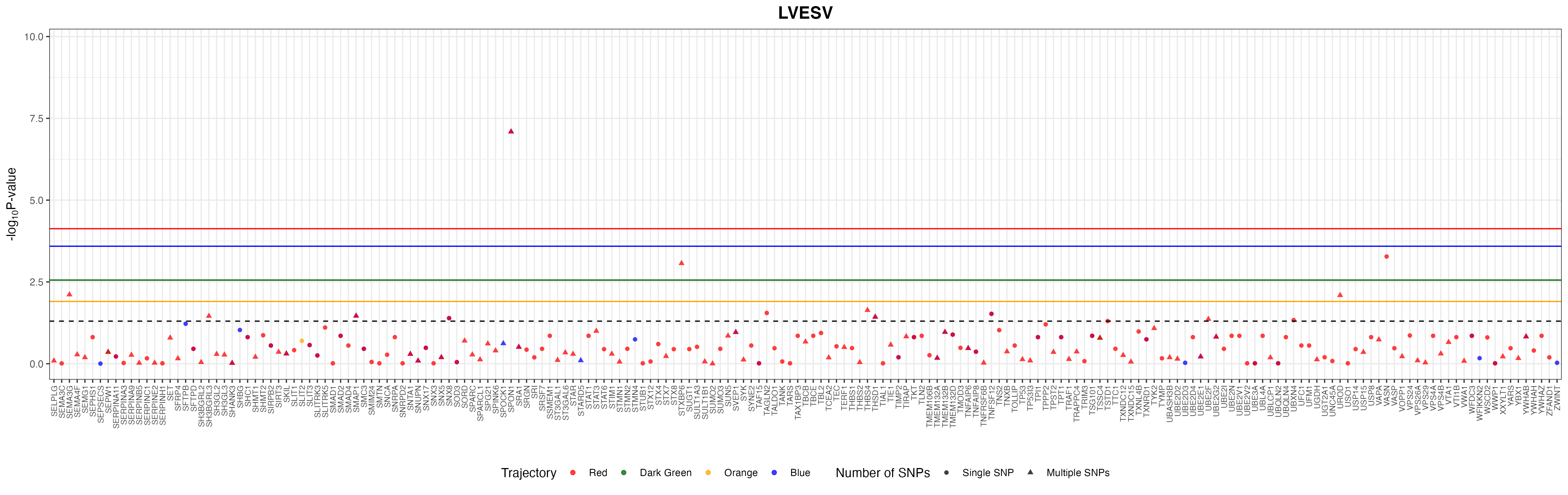


Abbreviations: LV, left ventricular; LVEDV, left ventricular end-diastolic volume; LVEF, left ventricular ejection fraction; LVESV, left ventricular end-systolic volume; MR, Mendelian Randomization; SNP, single-nucleotide polymorphism

**Supplemental Figure 2 (continued).** Manhattan plot of MR associations between trajectory associated proteins and LV volumes and LVEF


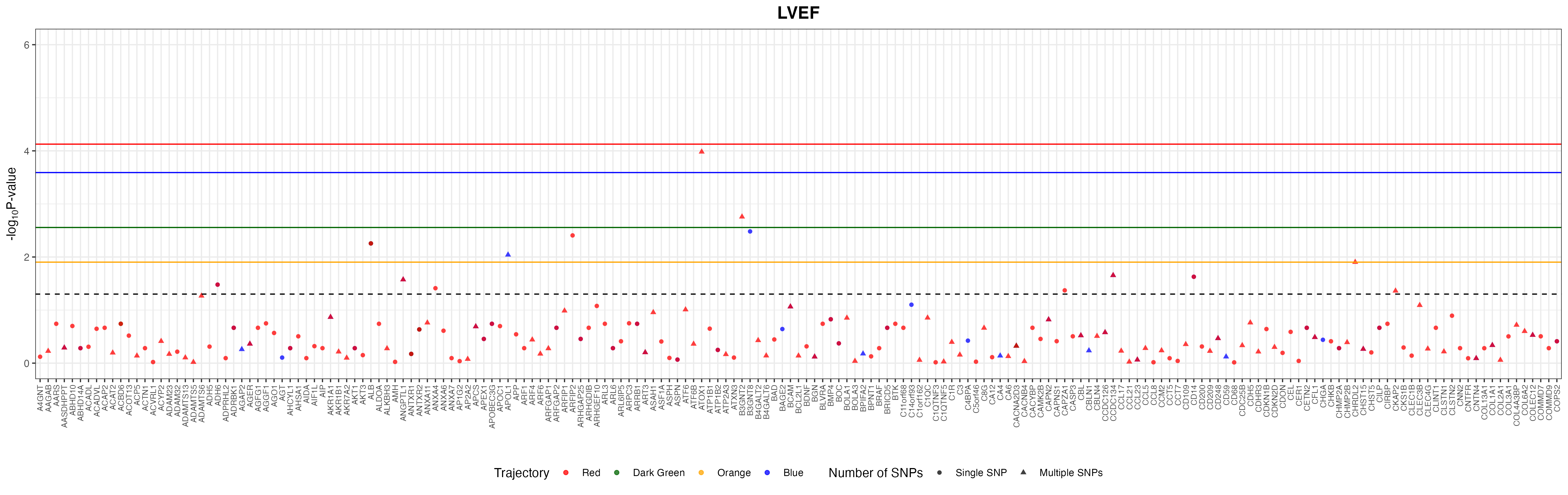


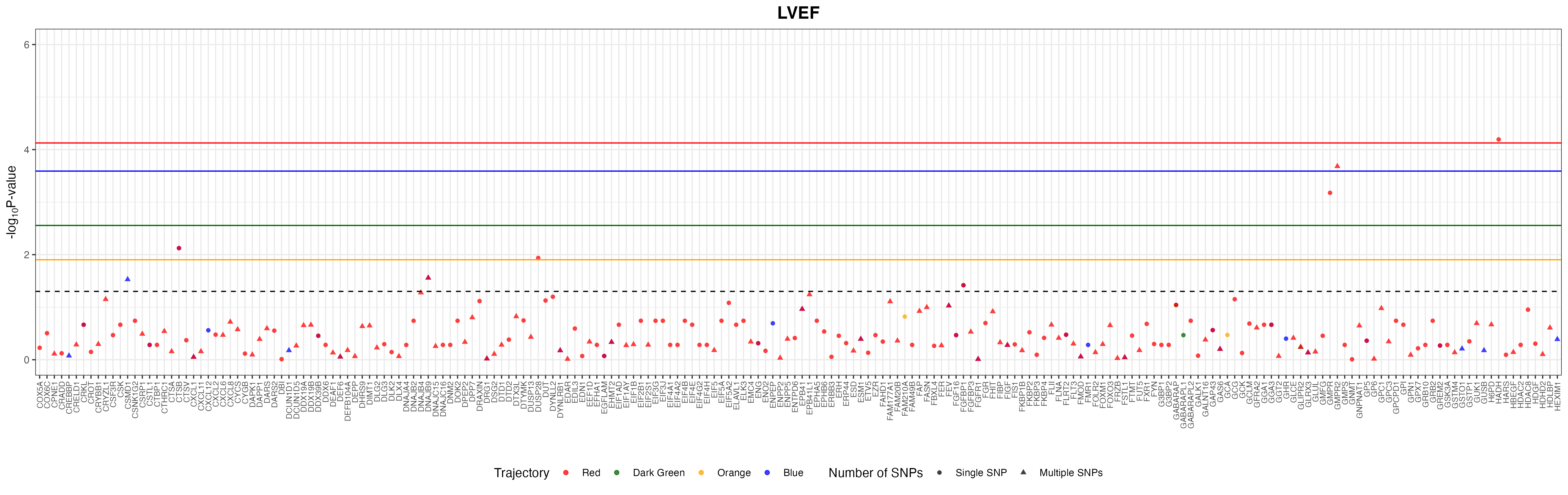


Abbreviations: LV, left ventricular; LVEDV, left ventricular end-diastolic volume; LVEF, left ventricular ejection fraction; LVESV, left ventricular end-systolic volume; MR, Mendelian Randomization; SNP, single-nucleotide polymorphism**Supplemental Figure 2 (continued).** Manhattan plot of MR associations between trajectory associated proteins and LV volumes and LVEF


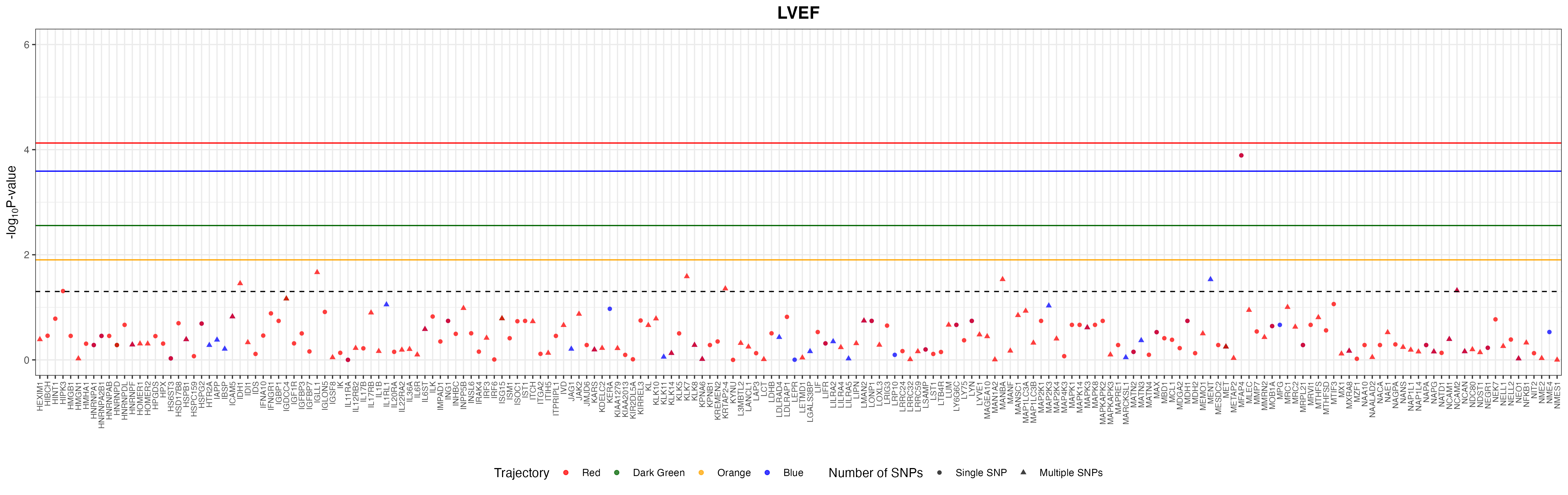


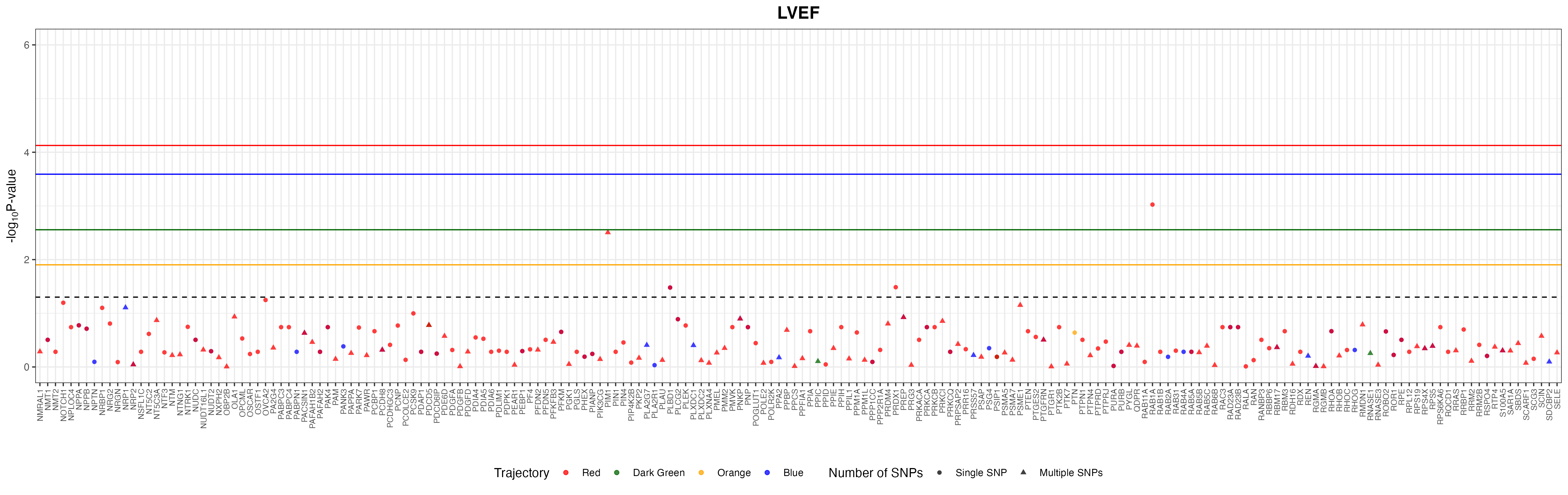


Abbreviations: LV, left ventricular; LVEDV, left ventricular end-diastolic volume; LVEF, left ventricular ejection fraction; LVESV, left ventricular end-systolic volume; MR, Mendelian Randomization; SNP, single-nucleotide polymorphism

**Supplemental Figure 2 (continued).** Manhattan plot of MR associations between trajectory associated proteins and LV volumes and LVEF


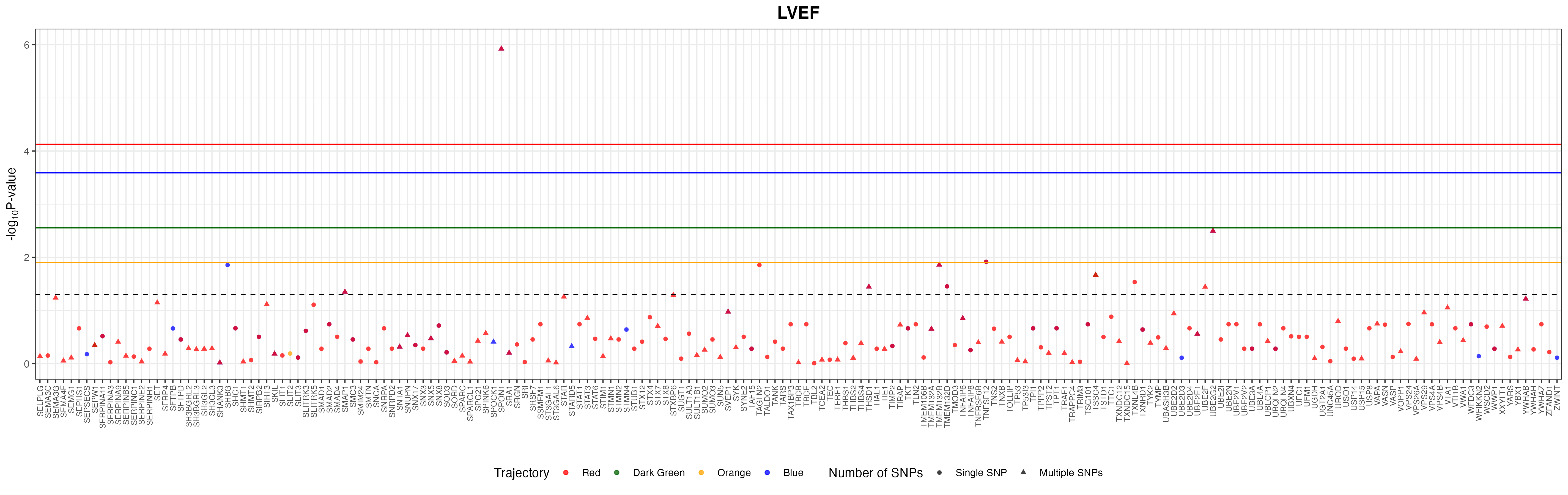


Abbreviations: LV, left ventricular; LVEDV, left ventricular end-diastolic volume; LVEF, left ventricular ejection fraction; LVESV, left ventricular end-systolic volume; MR, Mendelian Randomization; SNP, single-nucleotide polymorphism

**References**

1. Wright JD, Folsom AR, Coresh J, et al. The ARIC (Atherosclerosis Risk In Communities) Study. *J Am Coll Cardiol*. 2021;77:2939–2959.

2. Levey AS, Stevens LA, Schmid CH, et al. *A New Equation to Estimate Glomerular Filtration Rate*. 2009.

3. Loehr LR, Rosamond WD, Chang PP, Folsom AR, Chambless LE. Heart Failure Incidence and Survival (from the Atherosclerosis Risk in Communities Study). *Am J Cardiol*. 2008. Published online2008. https://doi.org/10.1016/j.amjcard.2007.11.061.

4. Rosamond WD, Chang PP, Baggett C, et al. Classification of heart failure in the atherosclerosis risk in communities (ARIC) study a comparison of diagnostic criteria. *Circ Heart Fail*. 2012;5:152–159.

5. Alonso A, Agarwal SK, Soliman EZ, et al. Incidence of atrial fibrillation in whites and African-Americans: The Atherosclerosis Risk in Communities (ARIC) study. *Am Heart J*. 2009;158:111–117.

6. Keku E, Rosamond W, Taylor HA, et al. Cardiovascular disease event classification in the Jackson Heart Study: methods and procedures. *Ethn Dis*. 2005;15:S6-62–70.

7. Gupta DK, Shah AM, Castagno D, et al. Heart Failure With Preserved Ejection Fraction in African Americans: The ARIC (Atherosclerosis Risk In Communities) Study. *JACC Heart Fail*. 2013;1:156–163.

8. White AD, Folsom AR, Chambless LE, et al. Community surveillance of coronary heart disease in the atherosclerosis risk in communities (ARIC) study: Methods and initial two years’ experience. *J Clin Epidemiol*. 1996;49:223–233.

9. Carpenter MA, Crow R, Steffes M, et al. *Laboratory, Reading Center, and Coordinating Center Data Management Methods in the Jackson Heart Study*.

10. Teichholz LE, Kreulen T, Herman M V, Gorlin R. Problems in echocardiographic volume determinations: echocardiographic-angiographic correlations in the presence of absence of asynergy. *Am J Cardiol*. 1976;37:7–11.

11. Shah AM, Cheng S, Skali H, et al. Rationale and Design of a Multicenter Echocardiographic Study to Assess the Relationship Between Cardiac Structure and Function and Heart Failure Risk in a Biracial Cohort of Community-Dwelling Elderly Persons: The Atherosclerosis Risk in Communities Study. *Circ Cardiovasc Imaging*. 2014;7:173–181.

12. Nagueh SF, Smiseth OA, Appleton CP, et al. Recommendations for the Evaluation of Left Ventricular Diastolic Function by Echocardiography: An Update from the American Society of Echocardiography and the European Association of Cardiovascular Imaging. *Journal of the American Society of Echocardiography*. 2016;29:277–314.

13. Parrinello CM, Grams ME, Couper D, et al. Recalibration of blood analytes over 25 years in the Atherosclerosis Risk in Communities Study: Impact of recalibration on chronic kidney disease prevalence and incidence. *Clin Chem*. 2015;61:938–947.

14. Gold L, Ayers D, Bertino J, et al. Aptamer-Based Multiplexed Proteomic Technology for Biomarker Discovery. *PLoS One*. 2010;5:e15004.

15. Ross JC, Castaldi PJ, Cho MH, et al. A Bayesian Nonparametric Model for Disease Subtyping: Application to Emphysema Phenotypes. *IEEE Trans Med Imaging*. 2017;36:343–354.

16. Watanabe S. Asymptotic Equivalence of Bayes Cross Validation and Widely Applicable Information Criterion in Singular Learning Theory. *J Mach Learn Res*. 2010;11:3571–3594.

17. Smith GD, Ebrahim S. “Mendelian randomization”: can genetic epidemiology contribute to understanding environmental determinants of disease? *Int J Epidemiol*. 2003;32:1–22.

18. Davey Smith G, Hemani G. Mendelian randomization: genetic anchors for causal inference in epidemiological studies. *Hum Mol Genet*. 2014;23:R89–R98.

19. Pietzner M, Wheeler E, Carrasco-Zanini J, et al. Mapping the proteo-genomic convergence of human diseases. *Science (1979)*. 2021;374:eabj1541.

20. Gudjonsson A, Gudmundsdottir V, Axelsson GT, et al. A genome-wide association study of serum proteins reveals shared loci with common diseases. *Nat Commun*. 2022;13:480.

21. Sun BB, Maranville JC, Peters JE, et al. Genomic atlas of the human plasma proteome. *Nature*. 2018;558:73–79.

22. Suhre K, Arnold M, Bhagwat AM, et al. Connecting genetic risk to disease end points through the human blood plasma proteome. *Nat Commun*. 2017;8:14357.

23. Ahola-Olli A V., Würtz P, Havulinna AS, et al. Genome-wide Association Study Identifies 27 Loci Influencing Concentrations of Circulating Cytokines and Growth Factors. *The American Journal of Human Genetics*. 2017;100:40–50.

24. Prins BP, Kuchenbaecker KB, Bao Y, et al. Genome-wide analysis of health-related biomarkers in the UK Household Longitudinal Study reveals novel associations. *Sci Rep*. 2017;7:11008.

25. Gilly A, Park Y-C, Png G, et al. Whole-genome sequencing analysis of the cardiometabolic proteome. *Nat Commun*. 2020;11:6336.

26. Folkersen L, Gustafsson S, Wang Q, et al. Genomic and drug target evaluation of 90 cardiovascular proteins in 30,931 individuals. *Nat Metab*. 2020;2:1135–1148.

27. Pirruccello JP, Bick A, Wang M, et al. Analysis of cardiac magnetic resonance imaging in 36,000 individuals yields genetic insights into dilated cardiomyopathy. *Nat Commun*. 2020;11:2254.

28. Bowden J, Davey Smith G, Burgess S. Mendelian randomization with invalid instruments: effect estimation and bias detection through Egger regression. *Int J Epidemiol*. 2015;44:512–25.

29. Hemani G, Zheng J, Elsworth B, et al. The MR-Base platform supports systematic causal inference across the human phenome. *Elife*. 2018;7.
